# Cannabis use and heart transplant listing: A survey of clinician practices

**DOI:** 10.1371/journal.pone.0310778

**Published:** 2024-12-12

**Authors:** Onyedika J. Ilonze, Shannon M. Knapp, Yelena Chernyak, Robert L. Page, LaKeisha J. Boyd, Sula Mazimba, Subha V. Raman, Chioma O. Enyi, Larry A. Allen, Khadijah Breathett

**Affiliations:** 1 Division of Cardiovascular Medicine, Indiana University, Indianapolis, IN, United States of America; 2 Department of Psychiatry, Indiana University, Indianapolis, IN, United States of America; 3 Department of Clinical Pharmacy, University of Colorado Skaggs School of Pharmacy and Pharmaceutical Sciences, Aurora, CO, United States of America; 4 Department of Biostatistics and Health Data Science, Indiana University School of Medicine and Richard M. Fairbanks School of Public Health, Indianapolis, IN, United States of America; 5 Division of Cardiology, University of Virginia, Charlottesville, VA, United States of America; 6 Division of Cardiology, Department of Medicine, University of Colorado School of Medicine, Aurora, CO, United States of America; Makerere University, UGANDA

## Abstract

No consensus exists for heart transplant listing for patients who use cannabis. We conducted a web-based survey to assess knowledge, and practice patterns towards patients with heart failure who use cannabis referred for transplant. A total of 140 clinicians (cardiologists (41.4%, n = 58), surgeons (7.1%, n = 10), pharmacists (9.3%, n = 13), advanced practice providers and coordinators) responded and responses were grouped by whether they responded that cannabis is “illegal in my state” (illegal), or “legal for medical and recreational use in my state,” (legal). There was a statistically significant difference in responses between the groups in the frequency of cannabis use that should preclude a patient from HT listing p = 0.0330) with respondents where cannabis is legal tending to answer that higher frequencies were acceptable. The groups in the “legal group” responded that a validated cannabis screening questionnaire could evaluate HT eligibility (p = 0.0111). A majority in the illegal group responding “No” as to whether their program allows pre- or post-transplant patients to use prescribed cannabis products (p < 0.0001). A majority in the illegal group responding “No” while the majority in the legal group responded “Yes” to “Does your HT center’s current selection criteria policy address medical cannabis use in potential transplant candidates?” (p = 0.0001). Health care providers generally agreed that a validated cannabis use disorder screening questionnaire would be useful and that 6 months of abstinence from cannabis is sufficient prior to HT listing. Significant heterogeneity exists regarding cannabis use as it relates to heart transplantation.

## Background

The acceptability and use of cannabis—both medically and recreationally—is growing. In the U.S., cannabis is legal in 38 of 50 states for medical use and 24 states for recreational use. At the federal level, cannabis is classified as a Schedule I drug under the Controlled Substances Act, determined to have a high potential for abuse and no accepted medical use, prohibiting its use for any purpose.

Uncertainty exists regarding heart transplant (HT) eligibility among patients with heart failure (HF) who use cannabis. Transplant societies have historically listed tobacco use and alcohol addiction/use disorder as contraindications to HT but provide limited guidance for patients who use cannabis and defer candidacy to individual centers [1]. Differing U.S. state cannabis legislations, inadequate knowledge about cannabis pharmacotherapy, and lack of safety data surrounding cannabis continues to worsen this uncertainty [2]. Key concerns by transplant clinicians regarding cannabis use include lack of outcomes data, concerns for poor medication adherence after transplantation, unpredictable variability in calcineurin inhibitor immunosuppressant levels (due to pharmacokinetic interaction with cannabis), concerns about cardiac allograft dysfunction, rejection, and fungal infections [3].

In a survey of HT programs, 59% allowed pre or post-HT patients to use medically prescribed cannabis products and 60% of programs required 6 months of abstinence prior to considering HT [4]. Another survey by Neyer et al. demonstrated that most respondents from states with laws prohibiting cannabis-using patients from being denied transplant listing reported denying all cannabis-using patients or mandating abstinence before transplant listing [5]. In a survey of the membership of a major transplant society, transplant centers varied in their approval processes for cannabis and transplant and there were differences regarding various organ types within the same institutions [6]. The purpose of this study was to assess the opinions and practice patterns of transplant clinicians and transplant center policies in relation to cannabis use in HT.

## Methods

**Fig 1:**
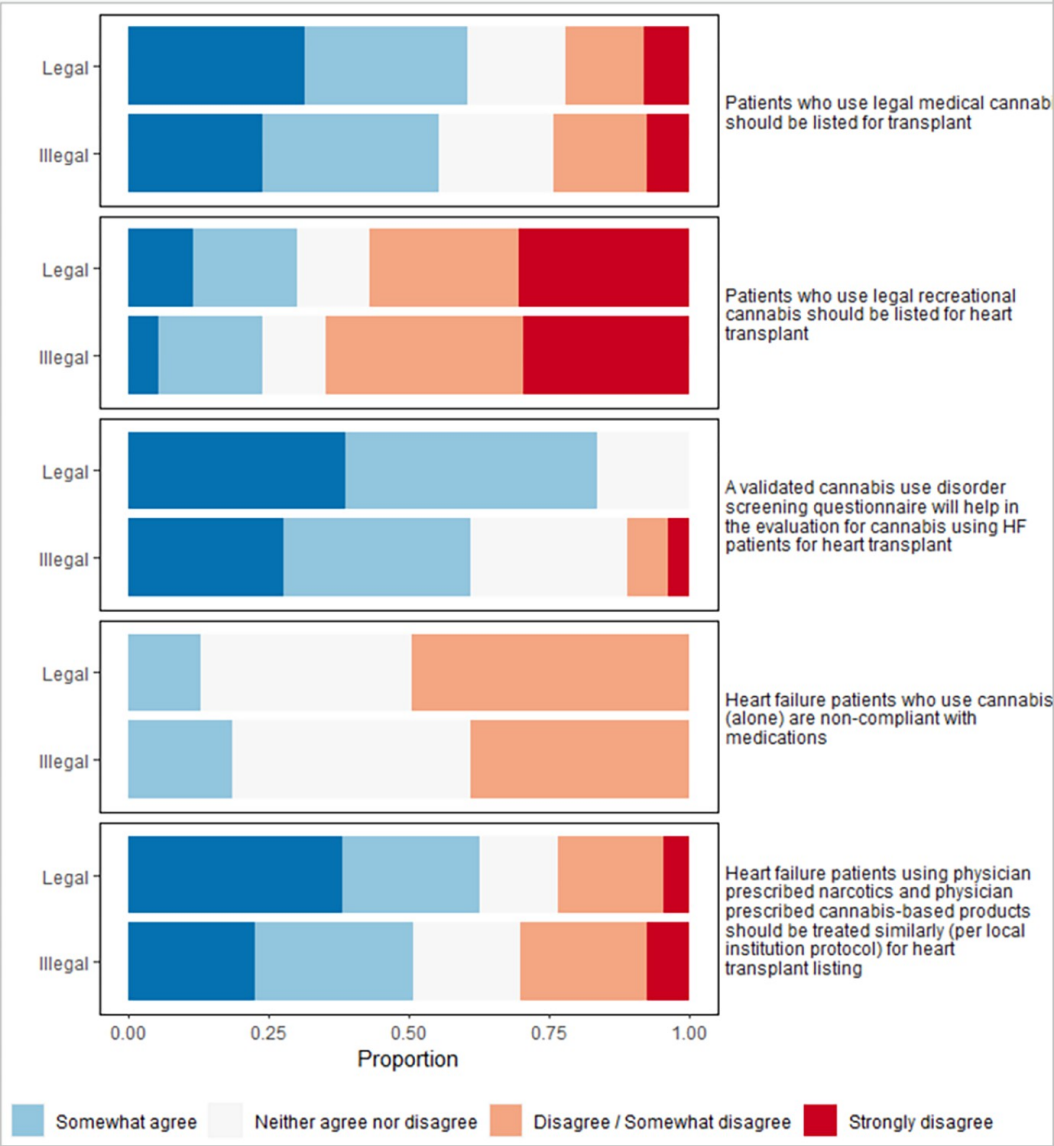
Key survey findings regarding cannabis use and heart transplant listing.

We conducted an anonymous, cross-sectional, web-based survey of HT healthcare professionals between December 18, 2021, and September 30, 2022. The survey was developed by the authorship team. Survey questions were developed based on the most important questions arising at transplant selection committee meetings and the questions were pre-tested once before survey administration to a group of HT clinicians. The final survey contained 16 optional multiple-choice questions **(Fig 1)**, and it could be submitted without completing all the questions. The survey assessed basic general demographic information including professional role, and self-identified gender. The data was analyzed using Microsoft Office 365 Excel software (Microsoft, Redmond, Washington, USA). The Indiana University Institutional Review Board approved the survey which contained questions designed to assess practice patterns for patients using cannabis referred for HT. A survey link was posted to the Heart Failure Society of America and the International Society of Heart and Lung Transplant websites. Respondents were asked to select whether cannabis is “illegal in my state”, “legal for medical and recreational use in my state,” or “legal for medical use in my state.” Those not responding to this question (n = 2) were excluded. We then tested for a difference in response between those in “illegal” versus “legal” states using Fisher’s exact test for nominal variables and the Brunner-Munzel test [7] for ordinal variables. Missing data for individual questions are reported but excluded from analysis. Informed consent was not obtained as it was a voluntary survey.

## Results

A total of 140 HT healthcare professionals were included in the analysis—cardiologists (41.4%, n = 58), surgeons (7.1%, n = 10), pharmacists (9.3%, n = 13), advanced practice providers and coordinators (23.6%, n = 33) Broken down by gender–females (60.7%, n = 85), males (33.6%, n = 47) and undisclosed (5.7%, n = 8) **(Table 1).** Their responses to the survey questions, grouped into 2 categories: i) illegal by whether they responded that cannabis is “illegal in my state/jurisdiction”, or ii) legal—“legal for medical and recreational use in my state/jurisdiction,” or “legal for medical use in my state/jurisdiction” with legal (n = 86; 61.4%) and illegal (n = 54; 38.6%) (**Table 1)**.

**Table 1:** Opinions regarding cannabis use and heart transplant listing.

Variable	Level	Illegal	Legal	P value
Q1: I am a	Cardiologist	20 (37.0%)	38 (44.2%)	0.2957^a^
	Other	15 (27.8%)	11 (12.8%)	
	Pharmacist	4 (7.4%)	9 (10.5%)	
	Surgeon	3 (5.6%)	7 (8.1%)	
	Transplant Coordinator	12 (22.2%)	21 (24.4%)	
Q3: In your opinion, what is the best way of assessing potential HT recipients for cannabis use?	Do not ask, do not tell policy	0 (0.0%)	2 (2.3%)	0.2075^a^
	History alone	1 (1.9%)	6 (7.0%)	
	History and toxicology	52 (96.3%)	78 (90.7%)	
	Other	1 (1.9%)	0 (0.0%)	
Q4: Patients who use legal medical cannabis should be listed for HT	Strongly disagree	4 (7.4%)	7 (8.1%)	0.4601^b^
	Somewhat disagree	9 (16.7%)	12 (14.0%)	
	Neither agree nor disagree	11 (20.4%)	15 (17.4%)	
	Somewhat agree	17 (31.5%)	25 (29.1%)	
	Strongly agree	13 (24.1%)	27 (31.4%)	
Q5: Patients who use legal recreational cannabis should be listed for HT?	Strongly disagree	16 (29.6%)	26 (30.2%)	0.4970^b^
	Somewhat disagree	19 (35.2%)	23 (26.7%)	
	Neither agree nor disagree	6 (11.1%)	11 (12.8%)	
	Somewhat agree	10 (18.5%)	16 (18.6%)	
	Strongly agree	3 (5.6%)	10 (11.6%)	
Q6 What frequency of cannabis use should preclude a patient from HT listing?	Any frequency of use is unacceptable	31 (57.4%)	34 (39.5%)	0.0330^b^
	Weekly	4 (7.4%)	3 (3.5%)	
	Daily	6 (11.1%)	16 (18.6%)	
	Multiple times per day	6 (11.1%)	21 (24.4%)	
	Any frequency of use is acceptable	6 (11.1%)	11 (12.8%)	
	Missing data	1 (1.9%)	1 (1.2%)	
Q7: Are you aware of any cannabis-use screening questionnaire to screen for cannabis use disorder?	No	50 (92.6%)	74 (86.0%)	0.4049^a^
	Yes	4 (7.4%)	11 (12.8%)	
	Missing data	0 (0.0%)	1 (1.2%)	
Q8: A validated cannabis use disorder screening questionnaire will help in the evaluation for cannabis using HF patients for HT eligibility?	Strongly disagree	2 (3.7%)	0 (0.0%)	0.0111^b^
	Somewhat disagree	4 (7.4%)	0 (0.0%)	
	Neither agree nor disagree	15 (27.8%)	14 (16.3%)	
	Somewhat agree	18 (33.3%)	38 (44.2%)	
	Strongly agree	15 (27.8%)	33 (38.4%)	
	Missing data	0 (0.0%)	1 (1.2%)	
Q9: Heart failure patients who use cannabis (alone) are non-compliant with medications?	Somewhat disagree	21 (38.9%)	42 (48.8%)	0.1979^b^
	Neither agree nor disagree	23 (42.6%)	32 (37.2%)	
	Somewhat agree	10 (18.5%)	11 (12.8%)	
	Missing data	0 (0.0%)	1 (1.2%)	
Q10: Do you feel comfortable accepting an excellent heart organ donor if the only contraindication is cannabis use for a potential heart transplant recipient?	Definitely not	5 (9.3%)	7 (8.1%)	0.8510^b^
	Probably not	7 (13.0%)	8 (9.3%)	
	Might or might not	6 (11.1%)	19 (22.1%)	
	Probably yes	19 (35.2%)	26 (30.2%)	
	Definitely yes	16 (29.6%)	25 (29.1%)	
	Missing data	1 (1.9%)	1 (1.2%)	
Q11: Does your program allow pre-transplant or post-transplant patients to use physician prescribed cannabis products?	No	43 (79.6%)	36 (41.9%)	<0.0001^b^
	Maybe	3 (5.6%)	25 (29.1%)	
	Yes	7 (13.0%)	25 (29.1%)	
	Missing data	1 (1.9%)	0 (0.0%)	
Q12: HF patients using physician prescribed narcotics and physician prescribed cannabis-based products should be treated similarly (per local institution protocol) for heart transplant listing?	Strongly disagree	4 (7.4%)	4 (4.7%)	0.0723^b^
	Disagree	12 (22.2%)	16 (18.6%)	
	Neither agree nor disagree	10 (18.5%)	12 (14.0%)	
	Somewhat agree	15 (27.8%)	21 (24.4%)	
	Strongly agree	12 (22.2%)	33 (38.4%)	
	Missing data	1 (1.9%)	0 (0.0%)	
Q13: Does your HT center’s current selection criteria policy address recreational cannabis use in potential transplant candidates?	No	14 (25.9%)	18 (20.9%)	0.7612^a^
	Other	2 (3.7%)	4 (4.7%)	
	Yes	36 (66.7%)	64 (74.4%)	
	Missing data	2 (3.7%)	0 (0.0%)	
Q14: Does your HT center’s current selection criteria policy address medical cannabis use in potential transplant candidates?	No	40 (74.1%)	37 (43.0%)	0.0001^a^
	Other	1 (1.9%)	2 (2.3%)	
	Yes	11 (20.4%)	47 (54.7%)	
	Missing data	2 (3.7%)	0 (0.0%)	
Q15: If your program considers patients who use cannabis for transplant, after what duration of cannabis abstinence are they considered for HT?	12 months	5 (9.3%)	3 (3.5%)	0.0025^a^
	6 months	42 (77.8%)	56 (65.1%)	
	Other	2 (3.7%)	10 (11.6%)	
	We transplant them anyway	1 (1.9%)	16 (18.6%)	
	Missing data	4 (7.4%)	1 (1.2%)	
Q16: I am	Female	37 (68.5%)	48 (55.8%)	0.2002^a^
	Male	15 (27.8%)	32 (37.2%)	
	Missing data	2 (3.7%)	6 (7.0%)	

^a^Fisher’s Exact test

^b^Brunner-Munzel test; HF–Heart Failure, HT–Heart Transplant

Most respondents 96.3% and 90.7% of the respondents in both the illegal and legal categories stated that a combination of history-taking and toxicology is the optimal modality to assess potential HT recipients for cannabis use respectively. (p = 0.2075). There was a statistically significant difference in responses between the groups in the frequency of cannabis use that should preclude a patient from HT listing (**Table 1**, Q6, p = 0.0330), with respondents where cannabis is legal tending to answer that higher frequencies were acceptable. The groups also differed in whether a validated cannabis use disorder screening questionnaire could evaluate HT eligibility in patients with cannabis use (**Table 1**, Q8, p = 0.0111), with those in the legal group tending to more towards agreement. As expected, there was a substantial difference between groups in response to whether their program allows pre- or post-transplant patients to use prescribed cannabis products (**Table 1**, Q11, p<0.0001), with a majority in the illegal group responding “No”. Similarly, there was a substantial difference between groups in response to “Does your HT center’s current selection criteria policy address medical cannabis use in potential transplant candidates?” (**Table 1**, Q14, p = 0.0001), with a majority in the illegal group responding “No” while the majority in the legal group responded “Yes”. In contrast, there was no difference between groups in response to having a policy on recreational cannabis use (**Table 1**, Q13, p = 0.7612). There was a significant difference between groups to the question “If your program considers patients who use cannabis for transplant, after what duration of cannabis abstinence are they considered for HT?” (**Table 1**, Q15, p = 0.0025) For both groups, most respondents selected “6 months;” notably more in the legal than the illegal group selected “We transplant then anyway” or “Other” while more in the illegal group than the legal group selected “12 months.” There was no statistically significant (α = 0.05) difference between groups in the remaining questions **(Table 1)**.

## Discussion

Our survey demonstrates the heterogeneity regarding cannabis use among HT team members, while comparing the respondents in states where cannabis is legal versus illegal. Respondents in states with legal cannabis believed that higher frequency of use is acceptable and that a validated cannabis questionnaire (Cannabis Use Disorders Identification Test-Revised survey) could identify problematic cannabis use even though most were unaware of this validated tool. Respondents in states where cannabis is illegal were less likely to allow prescribed cannabis products and to have policies surrounding medical cannabis use. Both groups agreed that 6 months of abstinence is appropriate to proceed with transplantation. The 6-month rule is arbitrary and is backed by expert consensus. Respondents believe that patients using prescribed opioids and cannabis should be considered similarly for HT. Respondents were wary of transplanting patients on long-term opioids for pain without tapering/considering less addictive analgesics. Our study found that respondents were not aware of a validated cannabis use disorder questionnaire that can be used prior to transplant. The difference in opinion in respondents from legal versus illegal cannabis use states reflects the heterogeneous attitudes of respondents. This suggests that legality of cannabis may influence respondent’s views on this subject.

Our paper adds on to an earlier work by Neyer et al. (2016) which found that most respondents supported HT listing for patients using legal medical cannabis [5] suggesting that attitudes remain unchanged. Our survey also illustrates a disconnect between the equivocal transplant guidelines in the face of increasing cannabis use and the uncertainty facing HT programs as they evaluate these patients for HT. Nevertheless, there is no data that pre-surgical cannabis use causes worse outcomes after transplantation. Some states have passed laws that prohibit transplant centers from denying transplant listing based solely on a patient’s use of medical cannabis [8].

Clinical outcomes data of cannabis use in other solid organ transplants has been inconsistent. A study of a national kidney transplant database to Medicare claims demonstrated that cannabis dependence or abuse was rare in kidney transplant recipients. Dependence or abuse in the year before transplant was not associated with death or graft failure in the year after transplant. However use in the first year posttransplant was associated with approximately 2-fold increased risk of death-censored graft failure, all-cause graft loss, and death (aHR, 1.79; 95% CL, 1.06–3.04) in the subsequent 2 years [9]. In a single center study of kidney transplant recipients, patients with isolated cannabis use had similar overall graft survival compared to nonusers (aHR 1.00, P = 0.994) [10]. Cannabis use disorder (abuse and dependence) is however associated with posttransplant psychosocial problems such as alcohol abuse, other drug abuse, noncompliance [9]. However, it must be noted that many patients assessed for HT use recreational cannabis which should be considered differently from medical cannabis.

## Limitations

This study has limitations. First, as a convenience survey it is at risk for responder and selection bias towards individuals more interested in HT cannabis use. Attitudes may be inadequately assessed by categorical responses which may not properly contextualize perception and practice. Finally, to preserve anonymity HT centers and their locations were not captured.

## Conclusion

Our study provides contemporary insight into the variability that exists among HT clinicians regarding cannabis use and heart transplantation. This highlights the need for further study to inform consistent guidance and recommendations across transplant centers to ensure standardized and equitable care for all transplant patients. This highlights the urgent need for development of data-driven approach regarding cannabis use and transplantation. These data-driven approaches should assess the impact of cannabis use on post-HT outcomes—allograft rejection and survival and accurately characterize the pharmacokinetic variability of the interaction between cannabis and calcineurin inhibitors. Finally, it is worth noting that the growing acceptance of cannabis in the US is ahead of nearly all other countries worldwide, such that better characterization of transplant eligibility in the US should be informative to countries who later follow the US example in relaxing cannabis laws.

## Supporting information

S1 File Marijuana statistical survey analysis. (PDF)

## Abbreviations

HT: heart transplantation
HF: heart failure
HFSA: Heart Failure Society of America
ISHLT: International Society of Heart and Lung Transplantation
CUD: Cannabis use disorder

## References

[1] Mehra MR, Canter CE, Hannan MM, Semigran MJ, Uber PA, Baran DA, et al. The 2016 International Society for Heart Lung Transplantation listing criteria for heart transplantation: A 10-year update. The Journal of heart and lung transplantation: the official publication of the International Society for Heart Transplantation. 2016;35(1):1–23. doi: 10.1016/j.healun.2015.10.023 26776864

[2] Ilonze OJ VD, Breathett K, Camacho-RIvera M, Raman V, Kobashigawa J, Allen LA. Cannabis Use and Heart Transplantation: Disparities and Opportunities to Improve Outcomes. Circulation: Heart Failure. 2022. 10.1161/CIRCHEARTFAILURE.122.009488 PMC9772032 36252094

[3] Melaragno JI, Bowman LJ, Park JM, Lourenco LM, Doligalski CT, Brady BL, et al. The Clinical Conundrum of Cannabis: Current Practices and Recommendations for Transplant Clinicians: An Opinion of the Immunology/Transplantation PRN of the American College of Clinical Pharmacy. Transplantation. 2021;105(2):291–9. doi: 10.1097/TP.0000000000003309 32413017

[4] Shah H, Fraser M, Agdamag AC, Maharaj V, Nzemenoh B, Martin CM, et al. Cardiac Transplantation and the Use of Cannabis. Life (Basel). 2021;11(10). doi: 10.3390/life11101063 34685434 PMC8539629

[5] Neyer J, Uberoi A, Hamilton M, Kobashigawa JA. Marijuana and Listing for Heart Transplant: A Survey of Transplant Providers. Circulation Heart failure. 2016;9(7). doi: 10.1161/CIRCHEARTFAILURE.115.002851 27413036

[6] Levi ME, Montague BT, Thurstone C, Kumar D, Huprikar SS, Kotton CN, et al. Marijuana use in transplantation: A call for clarity. Clinical transplantation. 2019;33(2):e13456. doi: 10.1111/ctr.13456 30506888

[7] Neuber K, Brunner E. Computational Statistics Data Analysis2007 June 2007.

[8] Jorgensen K. Legal Discrimination Against Organ Transplant Candidates: Medicinal Marijuana and the Double-Edged Sword. UIC J Marshall L Rev. 2019;52(3):859–80.

[9] Alhamad T, Koraishy FM, Lam NN, Katari S, Naik AS, Schnitzler MA, et al. Cannabis Dependence or Abuse in Kidney Transplantation: Implications for Posttransplant Outcomes. Transplantation. 2019;103(11):2373–82. doi: 10.1097/TP.0000000000002599 30747847 PMC6679817

[10] Fabbri KR, Anderson-Haag TL, Spenningsby AM, Israni A, Nygaard RM, Stahler PA. Marijuana use should not preclude consideration for kidney transplantation. Clinical transplantation. 2019;33(10):e13706. doi: 10.1111/ctr.13706 31498490

