# Supplementary material for: Cannabis use and heart transplant listing: A survey of clinician practices
Source: PLoS One. 2024 Dec 12;19(12):e0310778. doi: 10.1371/journal.pone.0310778 (PMC11637330; doi:10.1371/journal.pone.0310778)
Supplement: S1 File — (PDF) [file pone.0310778.s001.pdf]

Marijuana Survey Analysis
Version 1
Client: Dr. Onyedika Ilonze,

Shannon M. Knapp, Ph.D.
shaknapp@iu.edu

June 20, 2023

Contents

Contents **1**

1 Preliminaries (R) **3**

2 Notes on Methods **4**

3 Data **6**

3.1 Read-in Data File 6

3.2 Clean Data 9

3.3 Variable Key 12

4 Summary Statistics **14**

5 Analysis **17**

5.1 Ordinal Variables (Brunner-Munzel Tests) 17

5.2 Nominal Variables (Fisher's Exact Tests) 19

6 Figures - old **20**

7 Figures - current **32**

7.1 A: 5-level Agree-to-Disagree Scale 32

7.2 B: Yes/No (and Maybe/Other) 35

7.3 E (Q3-only) 37

7.4 C (Q6-only) 39

7.5 D (Q15-only) . . . 41

7.6 Combine Plots . . . 43

8 Appendix **44**

8.1 R Session Information . . . 45

8.2 Citations . . . 46

8.2.1 Citations (etc.) for Brunner-Munzel Test . . . 46

8.2.2 Citations for R and R-packages . . . 46

Chapter 1

Preliminaries (R)

```
>   library(xlsx)
>   library(ggplot2)
>   library(lawstat) ## has function brunner.munzel.test()
>   library(dplyr)
>   library(stringr)
>   library(janitor) ## Had function tabyl()
>   library(tidyr)
>   library(RColorBrewer)
```

```
>   ### Set the location "homedir" on the local machine
>     (homedir <- getwd() )

[1] "C:/Users/shaknapp/OneDrive - Indiana University/ProjectsIUmisc/IlonzeOnyedika/MarijuanaSurvey"

>   RPTNAME <- "MarijuanaSurveyRptV02"
>
>   DATADIR <- paste0(homedir,"/Data/")
>
>   OUTDIR <- "C:/Users/shaknapp/Indiana University/O365-BreathettStats - IlonzeOnyedika - IlonzeOnyedika/"
```

Prep File for Output to Excel

```
>   (timestamp = format(Sys.time(), "%Y-%m-%d %H:%M %Z"))

[1] "2023-06-20 07:50 PDT"

>   write.xlsx(timestamp, file = paste0(OUTDIR,RPTNAME,"output.xlsx"), sheetName="DateTime",
+              append=FALSE, col.names=FALSE, row.names = FALSE, showNA=FALSE)
```

Chapter 2

Notes on Methods

Note: this section is static; that is, it is not automatically updated with changes to the data/methods. This is current as of 12 June 2023.

Notes on data cleaning/processing.

- Version of data used was from 6 June 2023 (see Section 3.1)
- For analysis, I divided the sample into those who answered that Cannabis is "Illegal in my state" (Illegal) and those that answered "Legal for medical and recreational use in my state" or "Legal for medical use in my state" (Legal). Those that did not answer this question were *excluded* from the analysis (See Section 3.2). This got the final sample down to 141 observations.
- One observation was removed because this person answered *none* of the survey questions (See Section 3.2). Note that this person would have been excluded anyway because they did did not answer the Legal/Illegal question.
- For Q12, there were options for "Other" and "Neither agree nor disagree". In order to make this a proper ordinal variable, I lumped those two responses (in a Table, you might want to label this level "Other / Neither agree nor disagree", for the sake of transparency).
- For Q9, the first observation had the response = "2" which did not match any other options. This observation was changed to missing (NA)
- When I was looking over the Excel file with data, I noticed both *your* email address as well as Dr. Breathett's email address. I don't know if it is ethical to be counted as a subject (observation) in a survey/analysis for which you are an author. That strikes me as, at least, ethically questionable. However, I have not (at this point in time) removed those observations from the analysis. **As of 12 June 2023, I have excluded the observation with email address oilonze@iuhealth.org, this makes the final sample size 140 observations.**

Notes on analysis.

- For *ordinal* [1] variables, the Brunner-Munzel test (also known as the Generalized Wilcoxon test) was used to test for a difference in responses between groups (Legal/Illegal) in a way that accounts for the natural ordering of variables. See Section 8.2.1 for citations for this method. Please arrange a meeting with me (SMK) to discuss interpretation of these results.
- For *nominal* variables, Fisher's exact test was used to test for a difference in responses between groups.

[1] An ordinal variable is one where there is a natural ordering to the factor levels, for example: ("Strongly disagree", "Somewhat disagree","Neither agree nor disagree", "Somewhat agree", "Strongly agree"). This is in contrast to a *nominal* categorical variable, where there is no natural/inherent ordering of the levels, for example: (Cardiologist, Other,Pharmacist, Surgeon, Transplant Coordinator) or (Male, Female)

- The counts of those that did *not* answer a question were not included in the analyses (this should be made clear in the methods - especially if those that did not respond are shown in the Table); however, in the summary statistics (Section 4), those not answering are given (and counted in the percent over the whole group) - *if you want me to re-do the summary statistics to NOT count those that did not answer a question in the total percentage, please let me know.*
- I did not do any adjustments for multiple comparisons.

Please contact me if there is anything else you need for me to explain/clarify/etc.

Chapter 3

Data

3.1 Read-in Data File

The data file was sent to me via email on Tuesday, June 6, 2023 1:06 PM (PDT) by Onyedika Ilonze (oilonze@iu.edu)

This data file has 2 rows of headers and, thus, needs some cleaning and care to import.

Note: The original filename was: "Assessment of Heart Failure Patients who use Cannabis for Heart Transplant_May 23, 2022_11.14" but I was not able to read this is (I suspect due to the period in the file name) so I had to save the file under a new name (I made no changes to this file except for changing the name).
As of 20 June 2023 I'm not getting the error with the original file name (No idea what happened over the span of a few days...????)

```
>   filename.original <- "Assessment of Heart Failure Patients who use Cannabis for Heart Transplant_June 6, 2023_14.04"
>   file.mtime(paste0(DATADIR,filename.original,".xlsx"))

[1] "2023-06-20 06:11:57 PDT"

>   dat.xls0.original <- read.xlsx(file=paste0(DATADIR,filename.original,".xlsx"), sheetName = "Sheet0")
```

```
>   filename <- "DataRaw20230606"
>   file.mtime(paste0(DATADIR,filename,".xlsx"))

[1] "2023-06-06 13:24:19 PDT"
```

Showing the file as read in raw

```
>   dat.xls0 <- read.xlsx(file=paste0(DATADIR,filename,".xlsx"), sheetName = "Sheet0")
>   dim(dat.xls0)

[1] 144  40

>   names(dat.xls0)
```

```
 [1] "StartDate"             "EndDate"               "Status"                "IPAddress"
 [5] "Progress"              "Duration..in.seconds." "Finished"              "RecordedDate"
 [9] "ResponseId"            "RecipientLastName"     "RecipientFirstName"    "RecipientEmail"
[13] "ExternalReference"     "LocationLatitude"      "LocationLongitude"     "DistributionChannel"
[17] "UserLanguage"          "Q_RecaptchaScore"      "Q1"                    "Q1_5_TEXT"
[21] "Q2"                    "Q3"                    "Q3_5_TEXT"             "Q4"
[25] "Q5"                    "Q6"                    "Q7"                    "Q8"
[29] "Q9"                    "Q10"                   "Q11"                   "Q12"
[33] "Q12_6_TEXT"            "Q13"                   "Q13_3_TEXT"            "Q14"
[37] "Q14_3_TEXT"            "Q15"                   "Q15_4_TEXT"            "Q16"
```

```
>   dat.xls0[1:5,1:10]
```

```
        StartDate          EndDate         Status      IPAddress Progress Duration..in.seconds. Finished
1      Start Date         End Date  Response Type     IP Address Progress Duration (in seconds) Finished
2 44551.5700925926  44551.575787037     IP Address  71.245.164.18      100                   491     True
3 44551.7670138889 44551.7703819444     IP Address 208.38.250.141      100                   291     True
4 44554.7140740741 44554.7165393519     IP Address   68.226.55.33      100                   213     True
5 44567.5576157407 44567.5601851852 Survey Preview                     100                   221     True
      RecordedDate        ResponseId   RecipientLastName
1    Recorded Date       Response ID Recipient Last Name
2 44551.5757962037 R_1hZka1HboBmL2cR
3 44551.7704011806 R_10qt6gBY43UkTaB
4 44554.7165574306 R_3fGD0bjp9HaHQJz
5 44567.5601982176 R_erJboAGxIRXuUZH
```

```
>   AnalysisVarCols <- c(12,19,21,22,24:32,34,36,38,40)
>
>   dat0 <- read.xlsx(file=paste0(DATADIR,"DataRaw20230606.xlsx"), sheetIndex = 1,
+                     rowIndex=c(1,3:145),colIndex=AnalysisVarCols, stringsAsFactors=TRUE)
```

Note: in Q9 there was one response that was a “2” - I had Dr. Ilonze check this. He re-downloaded from Qualtrics and it was still there. I also asked about Q9 if there were only 3 choices (“Somewhat agree”, “Neither agree nor disagree”, and“Somewhat disagree”) or if “Strongly agree” and “Strongly disagree” were also choices and it was just the case that no one selected those. His response: “For question 9 I believe there were only three choices” (Tuesday, June 6, 2023 1:06 PM PDT).

```
>   summary(dat0)
```

```
                     RecipientEmail
                             :119
 abradford@iuhealth.org      :  1
 adrake6@iuhealth.org        :  1
 Albert.hicks3@gmail.com     :  1
 bforee@iuhealth.org         :  1
 bobhooker@email.arizona.edu:  1
 (Other)                     : 19
                                                                                                          Q1
                                                                                                          : 1
 Cardiologist - Heart Transplant or General Cardiology who takes care of HF and/or transplant patients:59
 Other                                                                                                 :27
 Pharmacist                                                                                            :13
 Surgeon                                                                                               :10
 Transplant Coordinator                                                                                :33

                                                  Q2                                 Q3
                                                   : 2                               :  1
 Illegal in my state                               :55   Do not ask, do not tell policy:  2
 Legal for medical and recreational use in my state:29   History alone                 :  7
 Legal for medical use in my state                 :57   History and toxicology        :132
```

```
                                               Other                        :  1


                       Q4                              Q5                                      Q6       Q7    
                        : 1                             : 1                                     : 3      :  2 
 Neither agree nor disagree:28   Neither agree nor disagree:17   Any frequency of use is acceptable  :17   No :126  
 Somewhat agree            :42   Somewhat agree            :26   Any frequency of use is unacceptable:66   Yes: 15  
 Somewhat disagree         :21   Somewhat disagree         :43   Daily                               :22            
 Strongly agree            :40   Strongly agree            :13   Multiple times per day              :28            
 Strongly disagree         :11   Strongly disagree         :43   Weekly                              : 7            

                       Q8                              Q9                      Q10        Q11     
                        : 2                             : 1                       : 3       : 3  
 Neither agree nor disagree:29   2                         : 1   Definitely not    :13   Maybe:28  
 Somewhat agree            :57   Neither agree nor disagree:57   Definitely yes    :41   No   :80  
 Somewhat disagree         : 4   Somewhat agree            :21   Might or might not:25   Yes  :32  
 Strongly agree            :48   Somewhat disagree         :63   Probably not      :15             
 Strongly disagree         : 3                                   Probably yes      :46             

                      Q12        Q13         Q14                           Q15                    Q16     
                        : 2        :  3        : 4                            :  6                    : 3  
 Disagree                  :28   No   : 32   No   :78   12 months             :  8   Female           :86  
 Neither agree nor disagree:18   Other:  6   Other: 3   6 months              :100   Male             :48  
 Other                     : 4   Yes  :102   Yes  :58   Other                 : 12   Prefer not to say: 6  
 Somewhat agree            :37                          We transplant them anyway: 17                        
 Strongly agree            :45                                                                               
 Strongly disagree         : 9                                                                               
```

The "2" in Q9 will have to be excluded as bad data.

There is also an "Other" option for Q12 that I will need to roll into the "Neither agree nor disagree" option to keep this as an Ordinal variable.

3.2 Clean Data

Need to convert most variables into *ordered* factors (with the proper ordering of levels) and also do the following.

- For Q1 shorten the response starting with “Cardiologist” to just be “Cardiologist
- For Q9 want the “2” to just be missing (NA)
- For Q12 lump the response “Other” into “Neither agree nor disagree”. Treat “Disagree” as same in ordering as “Somewhat disagree” (which was not an option in this question)
- For Q16 I want to make ”Prefer not to Say” just NA (Missing), otherwise it affects the Fisher’s Exact Test (new as of 20 June 2023)

```
>   Order1 <- c("Strongly disagree","Somewhat disagree","Neither agree nor disagree","Somewhat agree","Strongly agree")
>   Order2 <- c("Strongly disagree","Disagree","Neither agree nor disagree","Somewhat agree","Strongly agree")
>
>   dat1 <- dat0 %>%
+         ### Count how many questions were blank
+     mutate(NumBlank = rowSums(.=="" |is.na(.))) %>%
+     mutate(Q1 = ifelse(Q1=="",NA,
+                      ifelse(str_starts(Q1,"Cardio"),"Cardiologist",as.character(Q1))),
+            Q3 = factor(ifelse(Q3=="",NA,as.character(Q3))),
+            Q4 = factor(Q4, levels=Order1, ordered=TRUE),
+            Q5 = factor(Q5, levels=Order1, ordered=TRUE),
+            Q6 = factor(Q6,levels=c("Any frequency of use is unacceptable","Weekly","Daily","Multiple times per day",
+                                    "Any frequency of use is acceptable"),ordered=TRUE),
+            Q7 = factor(ifelse(Q7=="",NA,as.character(Q7))),
+            Q8 = factor(Q8,levels=Order1,ordered=TRUE),
+            Q9.original = Q9,
+            Q9 = factor(Q9, levels=c("Somewhat disagree","Neither agree nor disagree","Somewhat agree"), ordered=TRUE),
+            Q10 = factor(Q10, levels=c("Definitely not","Probably not","Might or might not","Probably yes","Definitely yes"), ordered=T[illegible]
+            Q11 = factor(Q11, levels=c("No","Maybe","Yes"), ordered = TRUE),
+            Q12.original = Q12,
+            Q12 = factor(ifelse(Q12=="Other","Neither agree nor disagree", as.character(Q12)), levels=Order2,ordered=TRUE),
+            Q13 = factor(ifelse(Q13=="",NA,as.character(Q13))),
+            Q14 = factor(ifelse(Q14=="",NA,as.character(Q14))),
+            Q15 = factor(ifelse(Q15=="",NA,as.character(Q15))),
+            Q16.original = Q16,
+            Q16 = factor(ifelse(Q16=="" | Q16=="Prefer not to say",NA,as.character(Q16))),
+            ) %>%
+     mutate(Legal = ifelse(Q2=="",NA,ifelse(str_starts(Q2,"Illegal"),"Illegal","Legal"))) %>%
+     mutate(one=1)
```

Breakdown of number of subjects that left 1, 2, 3, ... questions blank (note Q12 and Q12.fixed can be double counted)

```
>   dat1 %>% tabyl(NumBlank)


 NumBlank   n     percent
        0  24 0.167832168
        1 110 0.769230769
        2   3 0.020979021
        4   2 0.013986014
        5   2 0.013986014
        6   1 0.006993007
       17   1 0.006993007
```

Check observation with NumBlank ≥ 5

```
>   dat1 %>% filter(NumBlank >=5)


  RecipientEmail                     Q1                                Q2                    Q3
1                            Pharmacist               Illegal in my state History and toxicology
2                                  <NA>                                                     <NA>
3                                 Other Legal for medical use in my state History and toxicology
4                Transplant Coordinator               Illegal in my state History and toxicology
                          Q4                Q5                                   Q6   Q7                         Q8
1             Somewhat agree Strongly disagree Any frequency of use is unacceptable   No          Strongly disagree
2                       <NA>              <NA>                                 <NA> <NA>                       <NA>
3 Neither agree nor disagree Somewhat disagree                                 <NA> <NA>                       <NA>
4             Strongly agree Somewhat disagree                                 <NA>   No Neither agree nor disagree
                          Q9                Q10   Q11            Q12  Q13  Q14   Q15    Q16 NumBlank
1             Somewhat agree               <NA>  <NA>           <NA>  Yes   No  <NA>   <NA>        6
2                       <NA>               <NA>  <NA>           <NA> <NA> <NA>  <NA>   <NA>       17
3          Somewhat disagree Might or might not Maybe Somewhat agree  Yes  Yes Other   <NA>        5
4 Neither agree nor disagree       Probably yes    No Strongly agree <NA> <NA>  <NA> Female        5
                 Q9.original   Q12.original Q16.original   Legal one
1             Somewhat agree                             Illegal   1
2                                                           <NA>   1
3          Somewhat disagree Somewhat agree                Legal   1
4 Neither agree nor disagree Strongly agree       Female Illegal   1
```

I want to remove the person with ALL blank/missing responses

How many people didn't answer the Legal/Illegal Question?

```
>   dat1 %>% count(Q2)


                                                  Q2  n
1                                                      2
2                                Illegal in my state 55
3 Legal for medical and recreational use in my state 29
4                  Legal for medical use in my state 57


>   dat1 %>% count(Legal)


    Legal  n
1 Illegal 55
2   Legal 86
3    <NA>  2
```

FINALIZE data

- Remove observation with basically all responses missing
- Remove observations for which the question of legality was missing
- Removed observation with recipient email = "oilonze@iuhealth.org"

```
>   dat <- dat1 %>%
+     filter(NumBlank < 17) %>%
+     filter(!is.na(Legal)) %>%
+     filter(RecipientEmail != "oilonze@iuhealth.org")%>%
+     select(-RecipientEmail)
>
>   nrow(dat)
```

```
[1] 140
```

3.3 Variable Key

```
>   VarKey <- data.frame(t(dat.xls0[1,AnalysisVarCols ]), stringsAsFactors = FALSE)
```

```
>   for(i in 1:nrow(VarKey)){
+     cat("\n",rownames(VarKey)[i],"\n")
+     cat(writeLines(strwrap(VarKey$X1[i], width = 120)))
+   }
```

```
 RecipientEmail
Recipient Email

 Q1
I am a _____ - Selected Choice

 Q2
Cannabis is _____

 Q3
In your opinion, what is the best way of assessing potential heart transplant recipients for cannabis use? - Selected
Choice

 Q4
Patients who use legal medical cannabis should be listed for transplant

 Q5
Patients who use legal recreational cannabis should be listed for heart transplant?

 Q6
What frequency of cannabis use should preclude a patient from heart transplant listing?

 Q7
Are you aware of any cannabis-use screening questionnaire to screen for cannabis use disorder?

 Q8
A validated cannabis use disorder screening questionnaire will help in the evaluation for cannabis using HF patients
for heart transplant?

 Q9
Heart failure patients who use cannabis (alone) are non-compliant with medications?

 Q10
Do you feel comfortable accepting an excellent heart organ donor if the only contraindication is cannabis use for a
potential heart transplant recipient?

 Q11
Does your program allow pre-transplant or post-transplant patients to use physician prescribed cannabis products?

 Q12
Heart failure patients using physician prescribed narcotics and physician prescribed cannabis-based products should be
treated similarly (per local institution protocol) for heart transplant listing? - Selected Choice

 Q13
Does your heart transplant center's current selection criteria policy address recreational cannabis use in potential
transplant candidates? - Selected Choice

 Q14
Does your heart transplant center's current selection criteria policy address medicinal cannabis use in potential
transplant candidates? - Selected Choice

 Q15
If your program considers cannabis using HF patients for transplant, patients will be considered for transplant after
being cannabis-free for? - Selected Choice
```

Q16
I am

Chapter 4

Summary Statistics

Function to get Counts and Percentages by Group for Categorical Variable

```
>   SumStatsCatFUNC <- function(GRPVAR,VARLIST,DAT){
+     for(v in 1:length(VARLIST)){
+       tempvar = VARLIST[v]
+       smry.temp <-
+         DAT %>% tabyl(!!as.name(tempvar),!!as.name(GRPVAR)) %>%
+         adorn_totals("col") %>%
+         adorn_percentages("col") %>% adorn_pct_formatting(digits=1) %>%adorn_ns(position = "front") %>%
+         mutate(Variable = tempvar , Level=as.character(!!as.name(tempvar))) %>%
+         select(-!!as.name(tempvar))

+       if(v == 1){
+         my.summary <- smry.temp
+       } else{
+         my.summary <- bind_rows(my.summary, smry.temp )
+       }
+     }### end of for-loop (v)

+     my.summary <- my.summary %>% relocate(Variable, Level)

+     return(my.summary)

+   } ### end of function: SumStatsCatFUNC
```

```
>   SumStatsByLegal <-
+     SumStatsCatFUNC(GRPVAR = "Legal",
+                     VARLIST = c("one",intersect(names(dat0),names(dat)),"Q9.original","Q12.original","Q16.original"),
+                     DAT=dat)
```

Output to Excel

```
>   write.xlsx(as.data.frame(SumStatsByLegal),file = paste0(OUTDIR,RPTNAME,"output.xlsx"),
+              sheetName="SumStatsByLegal",
+              append=TRUE, col.names=TRUE, row.names = FALSE, showNA=FALSE)
```

```
>   SumStatsByLegal %>% print(.,row.names=FALSE)

 Variable                                          Level     Illegal       Legal        Total
      one                                              1 54 (100.0%) 86 (100.0%) 140 (100.0%)
       Q1                                   Cardiologist  20 (37.0%)  38 (44.2%)   58 (41.4%)
       Q1                                          Other  15 (27.8%)  11 (12.8%)   26 (18.6%)
       Q1                                     Pharmacist   4  (7.4%)   9 (10.5%)   13  (9.3%)
       Q1                                        Surgeon   3  (5.6%)   7  (8.1%)   10  (7.1%)
       Q1                         Transplant Coordinator  12 (22.2%)  21 (24.4%)   33 (23.6%)
       Q2                                                  0  (0.0%)   0  (0.0%)    0  (0.0%)
       Q2                            Illegal in my state 54 (100.0%)   0  (0.0%)   54 (38.6%)
       Q2 Legal for medical and recreational use in my state  0  (0.0%)  29 (33.7%)   29 (20.7%)
       Q2                  Legal for medical use in my state  0  (0.0%)  57 (66.3%)   57 (40.7%)
       Q3                    Do not ask, do not tell policy   0  (0.0%)   2  (2.3%)    2  (1.4%)
       Q3                                  History alone   1  (1.9%)   6  (7.0%)    7  (5.0%)
       Q3                         History and toxicology  52 (96.3%)  78 (90.7%)  130 (92.9%)
       Q3                                          Other   1  (1.9%)   0  (0.0%)    1  (0.7%)
       Q4                              Strongly disagree   4  (7.4%)   7  (8.1%)   11  (7.9%)
       Q4                              Somewhat disagree   9 (16.7%)  12 (14.0%)   21 (15.0%)
       Q4                     Neither agree nor disagree  11 (20.4%)  15 (17.4%)   26 (18.6%)
       Q4                                 Somewhat agree  17 (31.5%)  25 (29.1%)   42 (30.0%)
       Q4                                 Strongly agree  13 (24.1%)  27 (31.4%)   40 (28.6%)
       Q5                              Strongly disagree  16 (29.6%)  26 (30.2%)   42 (30.0%)
       Q5                              Somewhat disagree  19 (35.2%)  23 (26.7%)   42 (30.0%)
       Q5                     Neither agree nor disagree   6 (11.1%)  11 (12.8%)   17 (12.1%)
       Q5                                 Somewhat agree  10 (18.5%)  16 (18.6%)   26 (18.6%)
       Q5                                 Strongly agree   3  (5.6%)  10 (11.6%)   13  (9.3%)
       Q6          Any frequency of use is unacceptable  31 (57.4%)  34 (39.5%)   65 (46.4%)
       Q6                                         Weekly   4  (7.4%)   3  (3.5%)    7  (5.0%)
       Q6                                          Daily   6 (11.1%)  16 (18.6%)   22 (15.7%)
       Q6                        Multiple times per day   6 (11.1%)  21 (24.4%)   27 (19.3%)
       Q6            Any frequency of use is acceptable   6 (11.1%)  11 (12.8%)   17 (12.1%)
       Q6                                           <NA>   1  (1.9%)   1  (1.2%)    2  (1.4%)
       Q7                                             No  50 (92.6%)  74 (86.0%)  124 (88.6%)
       Q7                                            Yes   4  (7.4%)  11 (12.8%)   15 (10.7%)
       Q7                                           <NA>   0  (0.0%)   1  (1.2%)    1  (0.7%)
       Q8                              Strongly disagree   2  (3.7%)   0  (0.0%)    2  (1.4%)
       Q8                              Somewhat disagree   4  (7.4%)   0  (0.0%)    4  (2.9%)
       Q8                     Neither agree nor disagree  15 (27.8%)  14 (16.3%)   29 (20.7%)
       Q8                                 Somewhat agree  18 (33.3%)  38 (44.2%)   56 (40.0%)
       Q8                                 Strongly agree  15 (27.8%)  33 (38.4%)   48 (34.3%)
       Q8                                           <NA>   0  (0.0%)   1  (1.2%)    1  (0.7%)
       Q9                              Somewhat disagree  21 (38.9%)  42 (48.8%)   63 (45.0%)
       Q9                     Neither agree nor disagree  23 (42.6%)  32 (37.2%)   55 (39.3%)
       Q9                                 Somewhat agree  10 (18.5%)  11 (12.8%)   21 (15.0%)
       Q9                                           <NA>   0  (0.0%)   1  (1.2%)    1  (0.7%)
      Q10                                 Definitely not   5  (9.3%)   7  (8.1%)   12  (8.6%)
      Q10                                   Probably not   7 (13.0%)   8  (9.3%)   15 (10.7%)
      Q10                              Might or might not   6 (11.1%)  19 (22.1%)   25 (17.9%)
      Q10                                   Probably yes  19 (35.2%)  26 (30.2%)   45 (32.1%)
      Q10                                 Definitely yes  16 (29.6%)  25 (29.1%)   41 (29.3%)
      Q10                                           <NA>   1  (1.9%)   1  (1.2%)    2  (1.4%)
      Q11                                             No  43 (79.6%)  36 (41.9%)   79 (56.4%)
      Q11                                          Maybe   3  (5.6%)  25 (29.1%)   28 (20.0%)
      Q11                                            Yes   7 (13.0%)  25 (29.1%)   32 (22.9%)
      Q11                                           <NA>   1  (1.9%)   0  (0.0%)    1  (0.7%)
      Q12                              Strongly disagree   4  (7.4%)   4  (4.7%)    8  (5.7%)
      Q12                                       Disagree  12 (22.2%)  16 (18.6%)   28 (20.0%)
      Q12                     Neither agree nor disagree  10 (18.5%)  12 (14.0%)   22 (15.7%)
      Q12                                 Somewhat agree  15 (27.8%)  21 (24.4%)   36 (25.7%)
      Q12                                 Strongly agree  12 (22.2%)  33 (38.4%)   45 (32.1%)
      Q12                                           <NA>   1  (1.9%)   0  (0.0%)    1  (0.7%)
      Q13                                             No  14 (25.9%)  18 (20.9%)   32 (22.9%)
      Q13                                          Other   2  (3.7%)   4  (4.7%)    6  (4.3%)
      Q13                                            Yes  36 (66.7%)  64 (74.4%)  100 (71.4%)
      Q13                                           <NA>   2  (3.7%)   0  (0.0%)    2  (1.4%)
      Q14                                             No  40 (74.1%)  37 (43.0%)   77 (55.0%)
```

Q14	Other	1	(1.9%)	2	(2.3%)	3	(2.1%)
Q14	Yes	11	(20.4%)	47	(54.7%)	58	(41.4%)
Q14	<NA>	2	(3.7%)	0	(0.0%)	2	(1.4%)
Q15	12 months	5	(9.3%)	3	(3.5%)	8	(5.7%)
Q15	6 months	42	(77.8%)	56	(65.1%)	98	(70.0%)
Q15	Other	2	(3.7%)	10	(11.6%)	12	(8.6%)
Q15	We transplant them anyway	1	(1.9%)	16	(18.6%)	17	(12.1%)
Q15	<NA>	4	(7.4%)	1	(1.2%)	5	(3.6%)
Q16	Female	37	(68.5%)	48	(55.8%)	85	(60.7%)
Q16	Male	15	(27.8%)	32	(37.2%)	47	(33.6%)
Q16	<NA>	2	(3.7%)	6	(7.0%)	8	(5.7%)
Q9.original		0	(0.0%)	0	(0.0%)	0	(0.0%)
Q9.original	2	0	(0.0%)	1	(1.2%)	1	(0.7%)
Q9.original	Neither agree nor disagree	23	(42.6%)	32	(37.2%)	55	(39.3%)
Q9.original	Somewhat agree	10	(18.5%)	11	(12.8%)	21	(15.0%)
Q9.original	Somewhat disagree	21	(38.9%)	42	(48.8%)	63	(45.0%)
Q12.original		1	(1.9%)	0	(0.0%)	1	(0.7%)
Q12.original	Disagree	12	(22.2%)	16	(18.6%)	28	(20.0%)
Q12.original	Neither agree nor disagree	7	(13.0%)	11	(12.8%)	18	(12.9%)
Q12.original	Other	3	(5.6%)	1	(1.2%)	4	(2.9%)
Q12.original	Somewhat agree	15	(27.8%)	21	(24.4%)	36	(25.7%)
Q12.original	Strongly agree	12	(22.2%)	33	(38.4%)	45	(32.1%)
Q12.original	Strongly disagree	4	(7.4%)	4	(4.7%)	8	(5.7%)
Q16.original		1	(1.9%)	1	(1.2%)	2	(1.4%)
Q16.original	Female	37	(68.5%)	48	(55.8%)	85	(60.7%)
Q16.original	Male	15	(27.8%)	32	(37.2%)	47	(33.6%)
Q16.original	Prefer not to say	1	(1.9%)	5	(5.8%)	6	(4.3%)

Chapter 5

Analysis

5.1 Ordinal Variables (Brunner-Munzel Tests)

Brunner-Munzel Test is also known as the Generalized Wilcoxon test.

```
>   BMtest.FUNC <- function(VARLIST){

+     returndf <- data.frame(Variable=VARLIST)

+     ### Loop through the variables
+     for(var in 1:nrow(returndf)){

+       Legal <- subset(dat, Legal=="Legal")
+       Illegal <- subset(dat, Legal=="Illegal")

+       bmtest <- brunner.munzel.test(x=Legal[[returndf$Variable[var]]],
+                                     y=Illegal[[returndf$Variable[var]]],
+                                     alternative="two.sided")

+       returndf$RelativeEffect[var] <- bmtest$estimate
+       returndf$pval[var] <- round(bmtest$p.value,4)
+       returndf$n.Legal[var] <- sum(!is.na(Legal[[returndf$Variable[var]]]))
+       returndf$n.Illegal[var] <- sum(!is.na(Illegal[[returndf$Variable[var]]]))
+       returndf$ScaleSmallest[var] <- as.character(min(dat[[returndf$Variable[var]]], na.rm=TRUE))
+       returndf$ScaleBiggest[var] <- as.character(max(dat[[returndf$Variable[var]]], na.rm=TRUE))
+       returndf$BiggerValues[var] <- ifelse(bmtest$estimate > 0.5, "Illegal","Legal")
+       returndf$LegalIsMore[var] <- ifelse(bmtest$estimate < 0.5, as.character(max(dat[[returndf$Variable[var]]], na.rm=TRUE))
+                                            ,as.character(min(dat[[returndf$Variable[var]]], na.rm=TRUE)))
+     }### end of for-loop over variables (var)
+     return(returndf)
+   }### End of Function: BMtest.FUNC
>
>   BMtests <- BMtest.FUNC(VARLIST=c("Q4","Q5","Q6","Q8","Q9","Q10","Q11","Q12"))
```

Output to Excel

```
>   write.xlsx(BMtests,file = paste0(OUTDIR,RPTNAME,"output.xlsx"),
+              sheetName="BMtests",append=TRUE, col.names=TRUE, row.names = FALSE, showNA=FALSE)
```

```
>  print(BMtests)
```

	Variable	RelativeEffect	pval	n.Legal	n.Illegal	ScaleSmallest	ScaleBiggest	BiggerValues	LegalIsMore
1	Q4	0.4641473	0.4601	86	54	Strongly disagree	Strongly agree	Legal	Strongly agree
2	Q5	0.4673773	0.4970	86	54	Strongly disagree	Strongly agree	Legal	Strongly agree
3	Q6	0.3988901	0.0330	85	53	Any frequency of use is unacceptable	Any frequency of use is acceptable	Legal	Any frequency of use is acceptable
4	Q8	0.3761438	0.0111	85	54	Strongly disagree	Strongly agree	Legal	Strongly agree
5	Q9	0.5599129	0.1979	85	54	Somewhat disagree	Somewhat agree	Illegal	Somewhat disagree
6	Q10	0.5093230	0.8510	85	53	Definitely not	Definitely yes	Illegal	Definitely not
7	Q11	0.3146117	0.0000	86	53	No	Yes	Legal	Yes
8	Q12	0.4132295	0.0723	86	53	Strongly disagree	Strongly agree	Legal	Strongly agree

5.2 Nominal Variables (Fisher's Exact Tests)

```
>   FishTest.Func <- function(VARLIST){

+     returndf <- data.frame(Variable=VARLIST)

+     ### Loop through the variables
+     for(var in 1:nrow(returndf)){

+       returndf$pval[var] <-
+         round(
+           fisher.test(x=dat[[returndf$Variable[var]]],
+                       y=dat$Legal)$p.value
+           ,4)

+     }### End of for-loop over variables (var)

+     return(returndf)

+   }### End of Function: FishTest.FUNC
>
>   FisherTests <- FishTest.Func(VARLIST=c("Q1", "Q3", "Q7", "Q13", "Q14", "Q15", "Q16"))
```

```
>   print(FisherTests)
```

```
  Variable   pval
1       Q1 0.2957
2       Q3 0.2075
3       Q7 0.4049
4      Q13 0.7612
5      Q14 0.0001
6      Q15 0.0025
7      Q16 0.2002
```

Output to Excel

```
>   write.xlsx(FisherTests,file = paste0(OUTDIR,RPTNAME,"output.xlsx"),
+              sheetName="FisherTests",
+              append=TRUE, col.names=TRUE, row.names = FALSE, showNA=FALSE)
```

Chapter 6

Figures - old

Right now these are DRAFT versions of the Figures. If needed for publication, I can output the final version of the figure to a file (e.g., a JPEG (*.jpg) or BITMAP, (*.bmp)). Different publications have different requirements for figures, so please check figure requirements for wherever you plan to submit this to and let me know

Reformat data for these plots. Only Ordinal variables with the agree - disagree scale.

Note to make this all work in one figure I had to make all the levels of the variables consistent

- In Q4, Q5, Q8, there are 5 levels of response ("Strongly disagree","Somewhat disagree","Neither agree nor disagree","Somewhat agree","Strongly agree"), but in Q12 there is a level "Disagree" instead of "Somewhat disagree". To make everything consistent, I changed the second level to "Disagree / Somewhat disagree" for all of these questions.
- Q9 only had 3 levels, but I altered it to have the same 5 levels as the others (but no one can respond to the "Strongly" agree/disagree options as these did not exist)

```
>   Order1b <- c("Strongly disagree","Disagree / Somewhat disagree","Neither agree nor disagree","Somewhat agree","Strongly agree")
>
>   dat.fig1 <- dat %>%
+     select(Legal, Q4, Q5, Q8, Q9, Q12) %>%

+     ### Need to make the levels of the responses consistent
+     mutate(
+       Q4 = factor(Q4, levels=Order1, labels=Order1b, ordered=TRUE),
+       Q5 = factor(Q5, levels=Order1, labels=Order1b, ordered=TRUE),
+       Q8 = factor(Q8, levels=Order1, labels=Order1b, ordered=TRUE),
+       Q9 = factor(Q9, levels=Order1, labels=Order1b, ordered=TRUE),
+       Q12 = factor(Q12, levels=Order2, labels=Order1b, ordered=TRUE)
+     ) %>%

+     pivot_longer(cols=c("Q4", "Q5", "Q8","Q9","Q12"),
+                  names_to="Variable",
+                  values_to="Response",
+                  values_drop_na=TRUE) %>%

+     mutate(QuestionLabel = factor(Variable, levels=c("Q4","Q5","Q8","Q9","Q12"),
+                                   labels=c("Q4: List legal medical",
+                                            "Q5: List legal recreational",
+                                            "Q8: cannabis use disorder screening questionnaire",
+                                            "Q9: cannabis use = non-compliance",
+                                            "Q12: prescibed narcotics = prescibed cannabis"))) %>%
+     mutate(QuestionLabelLong = factor(Variable, levels=c("Q4","Q5","Q8","Q9","Q12"),
+                                   labels=c("Patients who use legal medical cannabis should be listed for transplant",
+                                            "Patients who use legal recreational cannabis should be listed for heart transplant",
```

```
+                                              "A validated cannabis use disorder screening questionnaire will help in the evaluation for
+                                              "Heart failure patients who use cannabis (alone) are non-compliant with medications",
+                                              "Heart failure patients using physician prescribed narcotics and physician prescribed canna
```

Version 1

```
>   dat.fig1 %>%
+     ggplot(aes(Legal)) +
+     geom_bar(aes(fill=Response),position = position_fill(reverse = TRUE))+

+     facet_wrap(~Variable, nrow=1)+


+     scale_fill_manual(values=brewer.pal(n=5, name="RdBu"), drop=FALSE, guide = guide_legend(reverse=TRUE))+
+     theme(panel.background=element_blank(),
+           panel.border = element_rect(fill=NA,color="black", linewidth=0.5, linetype="solid")) +
+     theme(strip.background = element_blank())+
+     #theme(legend.position = "bottom")+
+     theme(strip.text.y.right = element_text(angle = 0, hjust=0))+
+   labs(x="",fill="",y="Proportion")+
+     ggtitle("Version 1")
```

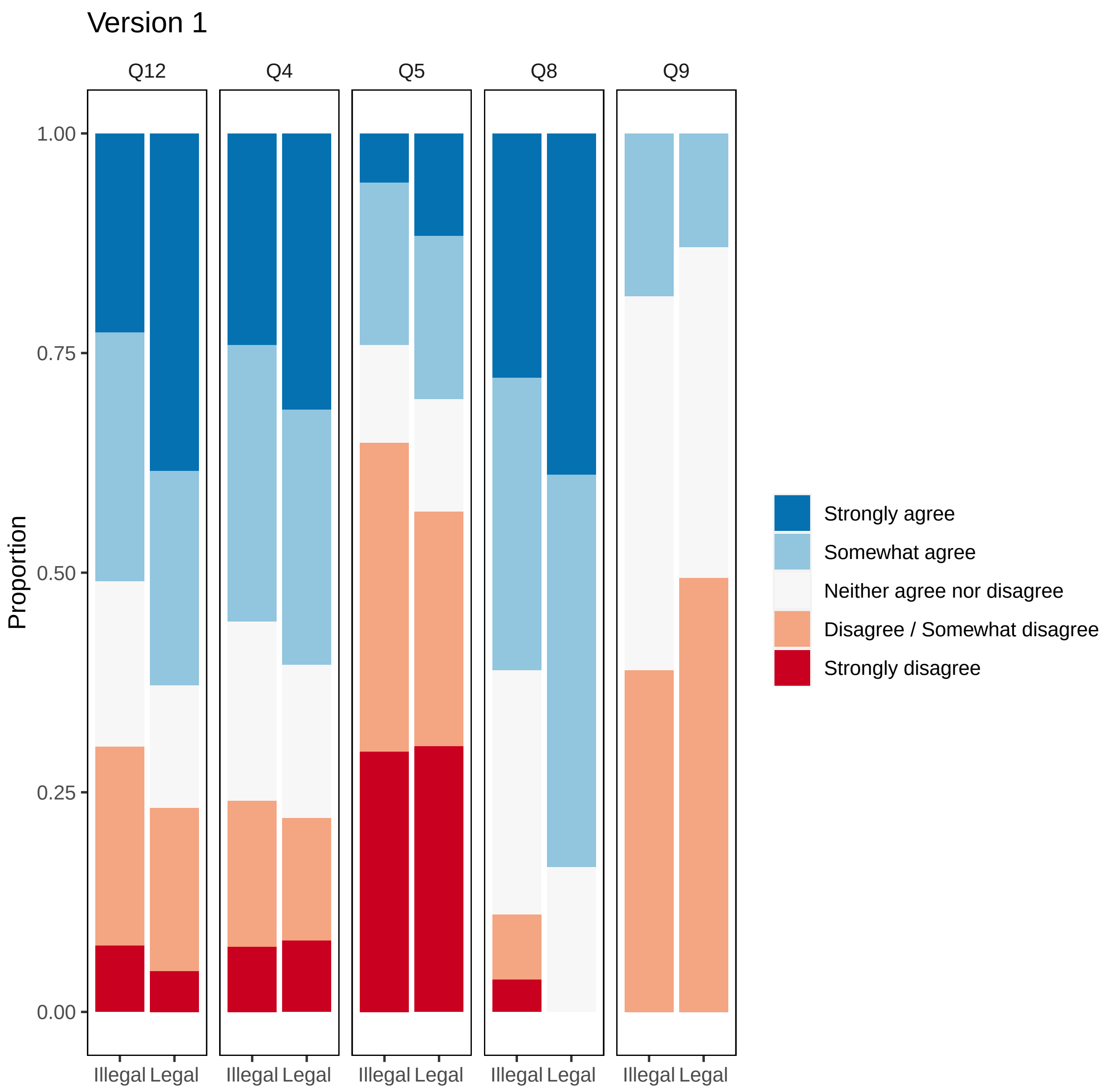

Version 1
Q12
Q4
Q5
Q8
Q9
Proportion
1.00
0.75
0.50
0.25
0.00
Illegal
Legal
Strongly agree
Somewhat agree
Neither agree nor disagree
Disagree / Somewhat disagree
Strongly disagree

Version 2

```
>   dat.fig1 %>%
+     ggplot(aes(Legal)) +
+     geom_bar(aes(fill=Response),position = position_fill(reverse = FALSE))+

+     facet_grid(QuestionLabel~.,  labeller = label_wrap_gen(width = 40, multi_line = TRUE))+
+     coord_flip()+


+     scale_fill_manual(values=brewer.pal(n=5, name="RdBu"), drop=FALSE, guide = guide_legend(reverse=TRUE,nrow=2,byrow=TRUE))+
+     theme(panel.background=element_blank(),
+           panel.border = element_rect(fill=NA,color="black", linewidth=0.5, linetype="solid")) +
+     theme(strip.background = element_blank())+
+     theme(legend.position = "bottom")+
+     theme(strip.text.y.right = element_text(angle = 0, hjust=0))+
+   labs(x="",fill="",y="Proportion")+
+     ggtitle("Version 2")
```

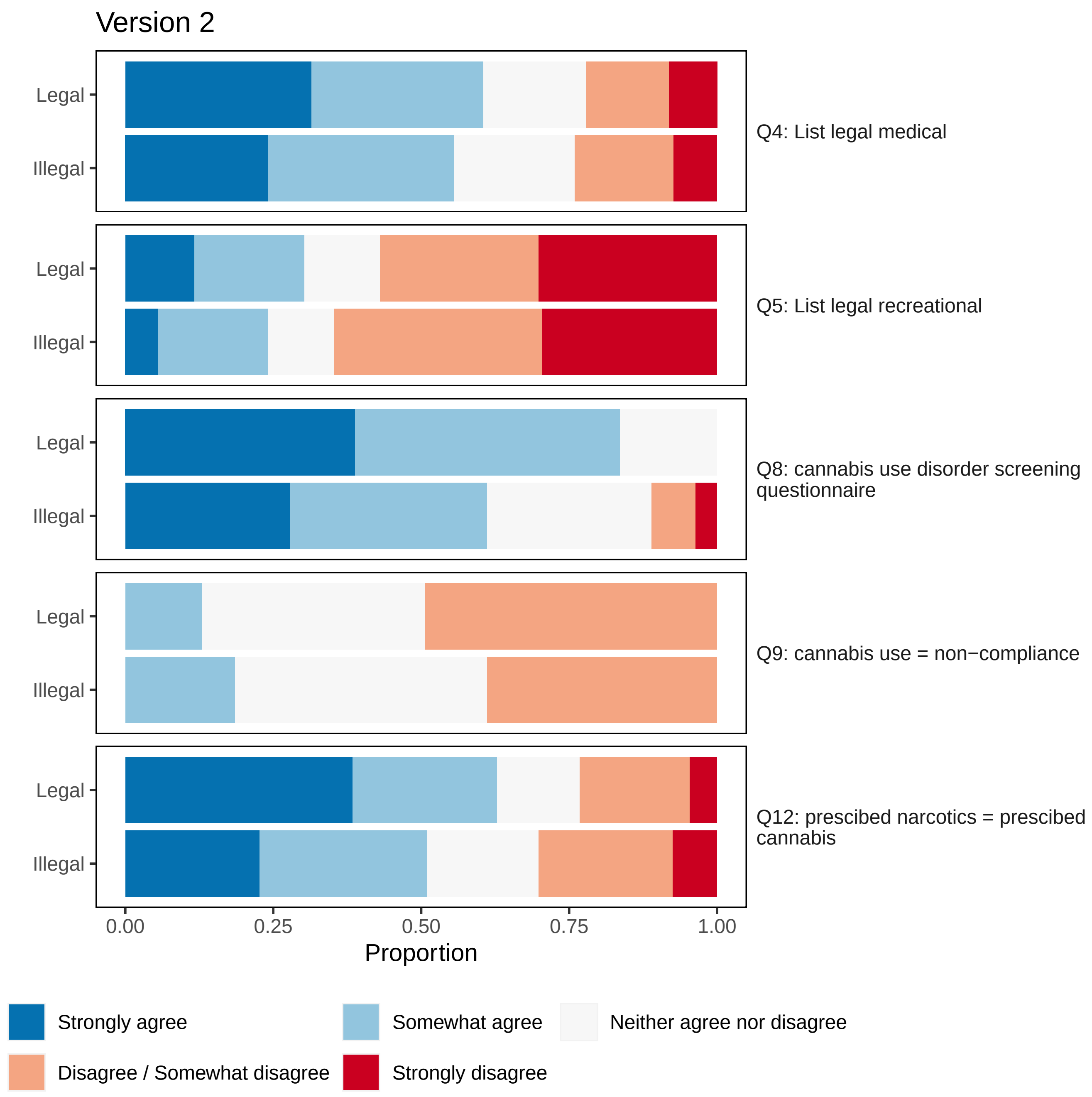
Version 2
Legal
Illegal
Q4: List legal medical
Legal
Illegal
Q5: List legal recreational
Legal
Illegal
Q8: cannabis use disorder screening questionnaire
Legal
Illegal
Q9: cannabis use = non−compliance
Legal
Illegal
Q12: prescibed narcotics = prescibed cannabis
0.00
0.25
0.50
0.75
1.00
Proportion
Strongly agree
Somewhat agree
Neither agree nor disagree
Disagree / Somewhat disagree
Strongly disagree

Version 3a

```
>   dat.fig1 %>%
+     ggplot(aes(y=Legal)) +
+     geom_bar(aes(fill=Response),position = "fill")+

+     facet_grid(QuestionLabelLong~.,  labeller = label_wrap_gen(width = 40, multi_line = TRUE))+


+     scale_fill_manual(values=brewer.pal(n=5, name="RdBu"), drop=FALSE, guide = guide_legend(reverse=TRUE,nrow=2,byrow=TRUE))+
+     theme(panel.background=element_blank(),
+           panel.border = element_rect(fill=NA,color="black", linewidth=0.5, linetype="solid")) +
+     theme(strip.background = element_blank())+
+     theme(legend.position = "bottom")+
+     theme(strip.text.y.right = element_text(angle = 0, hjust=0))+
+   labs(x="Proportion",fill="",y="")+
+     ggtitle("Version 3a")
```

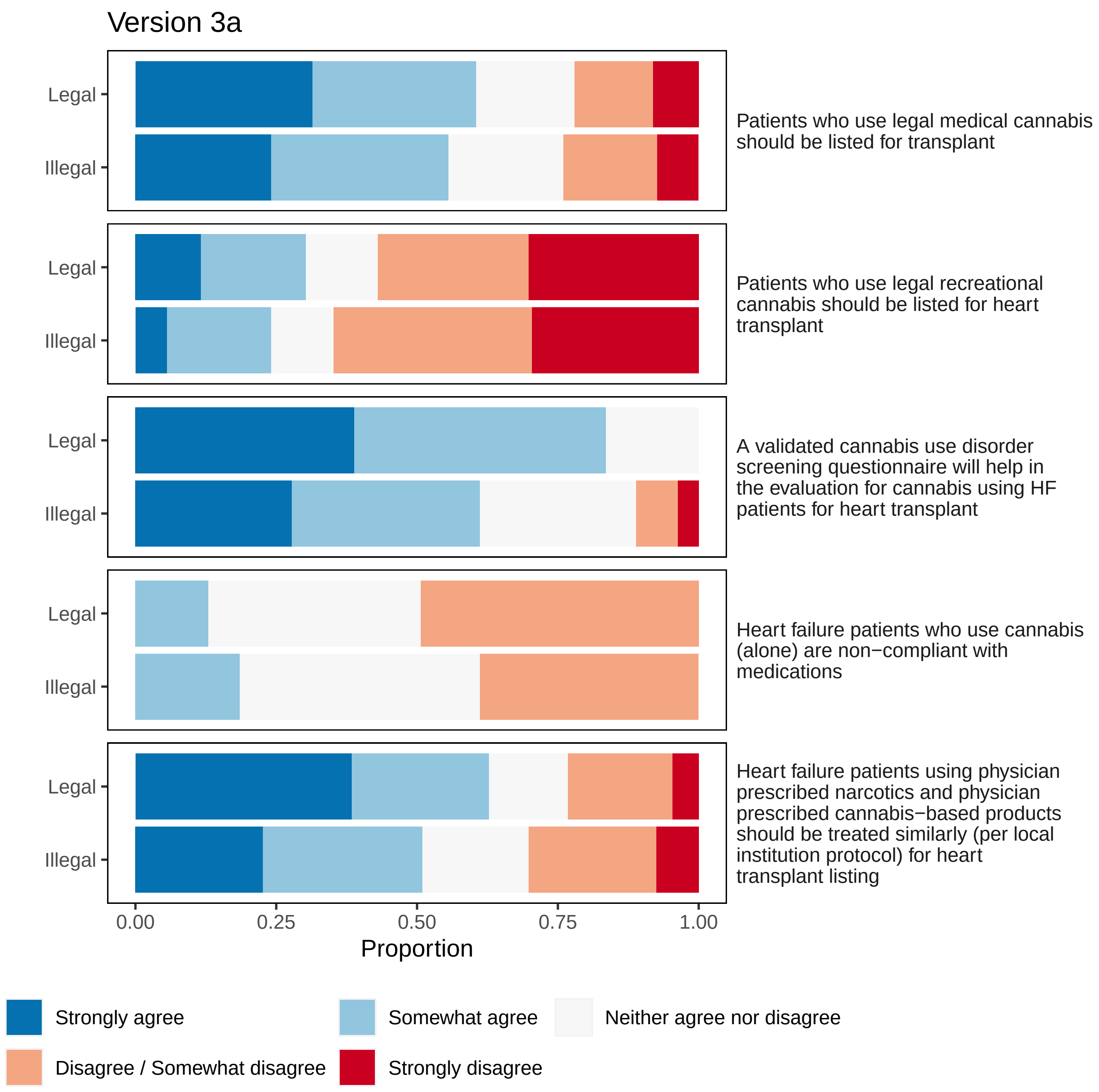
Version 3a
Legal
Illegal
Patients who use legal medical cannabis should be listed for transplant
Legal
Illegal
Patients who use legal recreational cannabis should be listed for heart transplant
Legal
Illegal
A validated cannabis use disorder screening questionnaire will help in the evaluation for cannabis using HF patients for heart transplant
Legal
Illegal
Heart failure patients who use cannabis (alone) are non−compliant with medications
Legal
Illegal
Heart failure patients using physician prescribed narcotics and physician prescribed cannabis−based products should be treated similarly (per local institution protocol) for heart transplant listing
0.00
0.25
0.50
0.75
1.00
Proportion
Strongly agree
Somewhat agree
Neither agree nor disagree
Disagree / Somewhat disagree
Strongly disagree

Version 3b

```
>   dat.fig1 %>%
+     ggplot(aes(y=Legal)) +
+     geom_bar(aes(fill=Response),position = "fill")+

+     facet_grid(QuestionLabelLong~.,  labeller = label_wrap_gen(width = 40, multi_line = TRUE))+


+     scale_fill_manual(values=brewer.pal(n=5, name="RdBu"), drop=FALSE, guide = guide_legend(reverse=TRUE,label.position = "bottom"))+
+     theme(panel.background=element_blank(),
+           panel.border = element_rect(fill=NA,color="black", linewidth=0.5, linetype="solid")) +
+     theme(strip.background = element_blank())+
+     theme(legend.position = "bottom")+
+     theme(legend.direction="horizontal",legend.text=element_text(angle=90,hjust=1))+
+     #theme(legend.direction="horizontal",legend.text.align=0)+
+     theme(strip.text.y.right = element_text(angle = 0, hjust=0))+
+   labs(x="Proportion",fill="",y="")+
+     ggtitle("Version 3b")
```

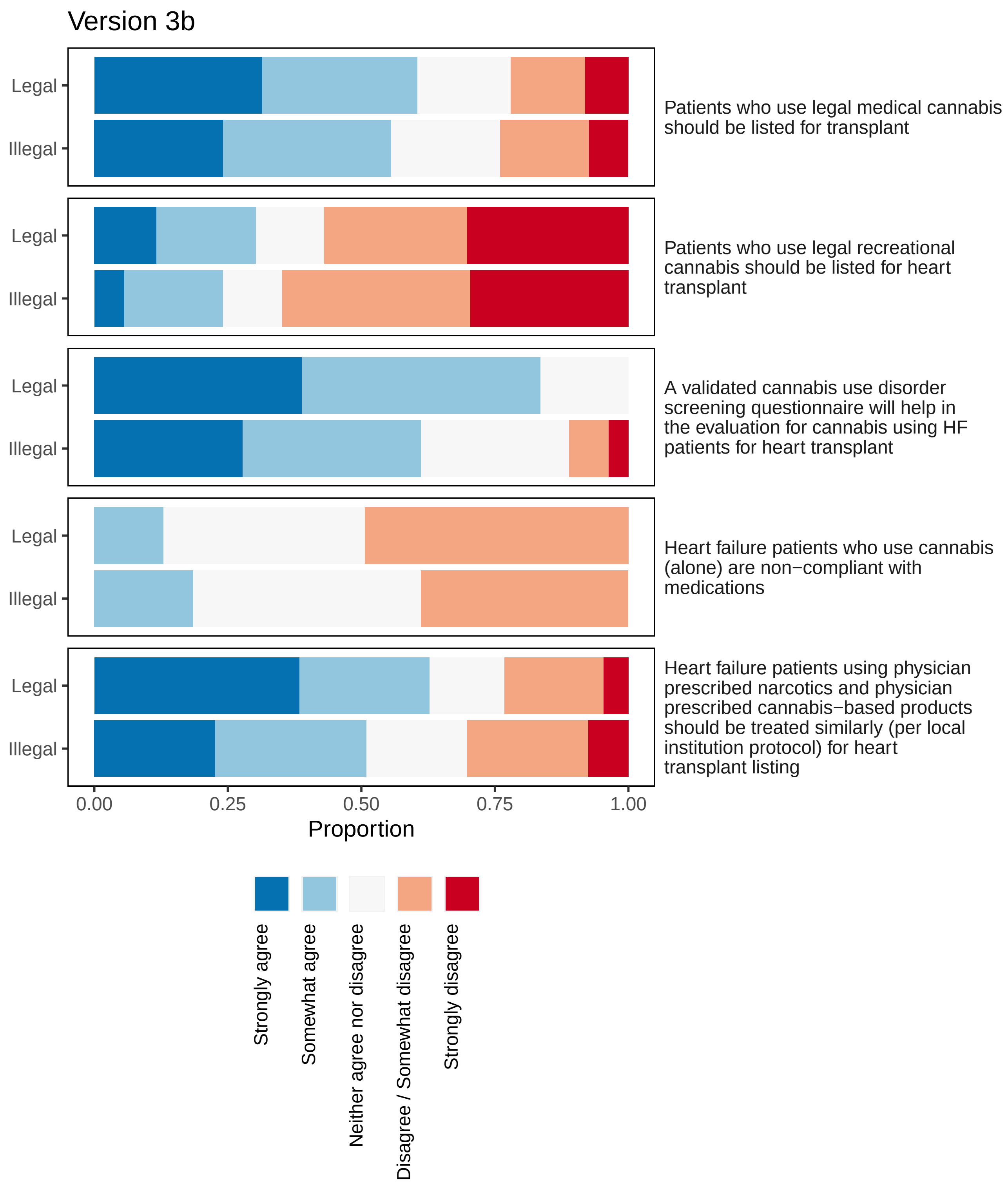

Version 3b
Legal
Illegal
Patients who use legal medical cannabis should be listed for transplant
Legal
Illegal
Patients who use legal recreational cannabis should be listed for heart transplant
Legal
Illegal
A validated cannabis use disorder screening questionnaire will help in the evaluation for cannabis using HF patients for heart transplant
Legal
Illegal
Heart failure patients who use cannabis (alone) are non−compliant with medications
Legal
Illegal
Heart failure patients using physician prescribed narcotics and physician prescribed cannabis−based products should be treated similarly (per local institution protocol) for heart transplant listing
0.00
0.25
0.50
0.75
1.00
Proportion
Strongly agree
Somewhat agree
Neither agree nor disagree
Disagree / Somewhat disagree
Strongly disagree

Version 4

```
>   dat.fig1 %>%
+     ggplot(aes(x=Legal)) +
+     geom_bar(aes(fill=Response),position = "fill")+

+     facet_wrap(~QuestionLabelLong,  nrow=1,labeller = label_wrap_gen(width = 40, multi_line = TRUE))+


+     scale_fill_manual(values=brewer.pal(n=5, name="RdBu"), drop=FALSE, guide = guide_legend(reverse=FALSE))+
+     theme(panel.background=element_blank(),
+           panel.border = element_rect(fill=NA,color="black", linewidth=0.5, linetype="solid")) +
+     theme(strip.background = element_blank())+
+     theme(legend.position = "right")+
+     theme(strip.text = element_text(angle = 90, hjust=0))+
+   labs(y="Proportion",fill="",x="")+
+     ggtitle("Version 4")
```

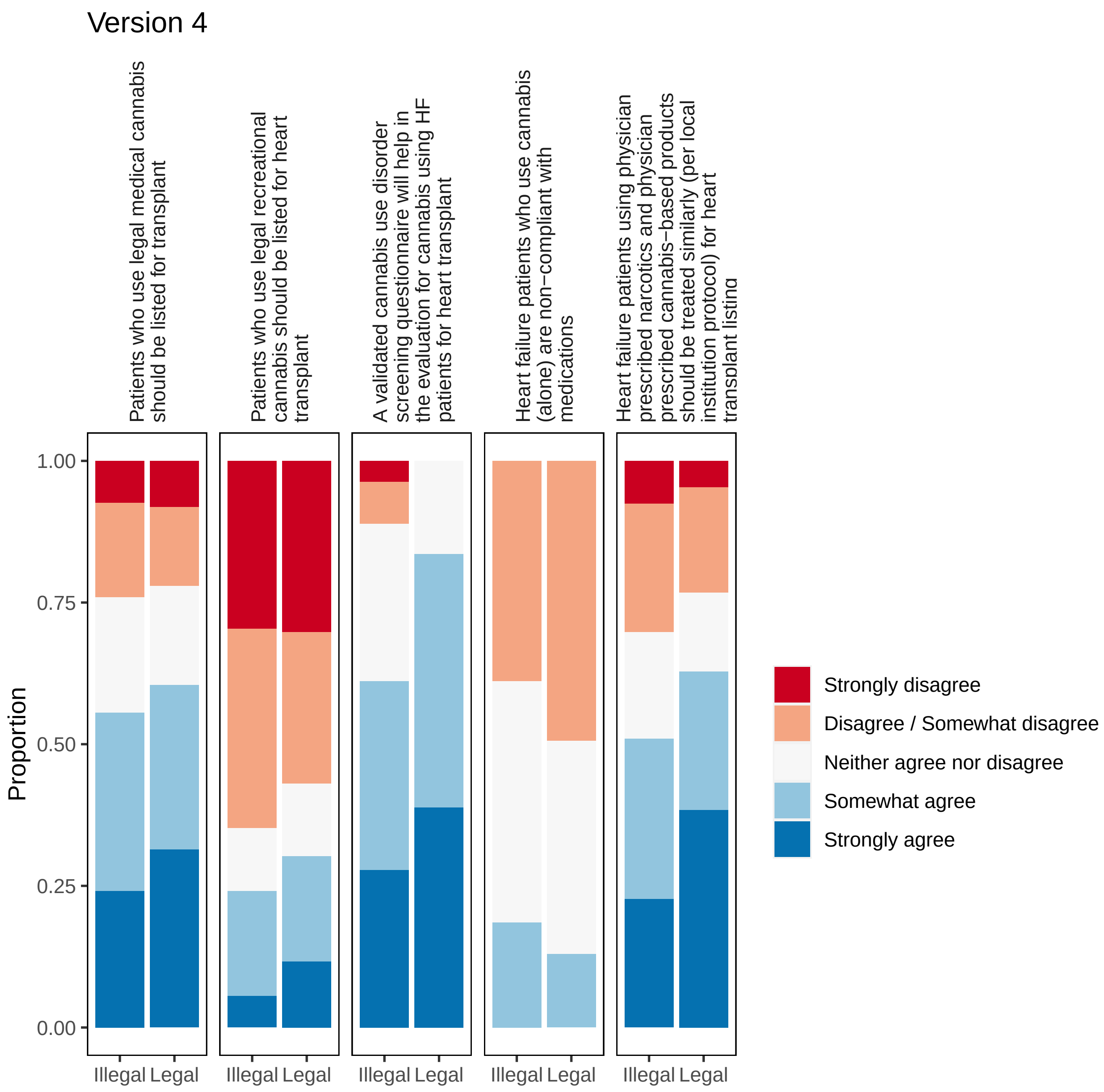

Version 4
Patients who use legal medical cannabis should be listed for transplant
Patients who use legal recreational cannabis should be listed for heart transplant
A validated cannabis use disorder screening questionnaire will help in the evaluation for cannabis using HF patients for heart transplant
Heart failure patients who use cannabis (alone) are non−compliant with medications
Heart failure patients using physician prescribed narcotics and physician prescribed cannabis−based products should be treated similarly (per local institution protocol) for heart transplant listing
Proportion
1.00
0.75
0.50
0.25
0.00
Illegal
Legal
Strongly disagree
Disagree / Somewhat disagree
Neither agree nor disagree
Somewhat agree
Strongly agree

Chapter 7

Figures - current

Right now these are DRAFT versions of the Figures. If needed for publication, I can output the final version of the figure to a file (e.g., a JPEG (*.jpg) or BITMAP, (*.bmp)). Different publications have different requirements for figures, so please check figure requirements for wherever you plan to submit this to and let me know

See notes about the plots at the top of each section. In the interest of minimizing the number of stand-alone plots, I had to make modifications. These modificaitons should be noted in the figure caption/footnote. To omit some of these details would be MISLEADING. For example, Q9 only had options for 3 levels: ("Somewhat disagree","Neither agree nor disagree","Somewhat agree") but plotting this with Q4, Q5, Q8, and Q12 makes it look there were 5 options for response (i.e., that "Strongly agree" and "Strongly disagree" were options when they were not). Something similar was done with Q7 (only "Yes" and "No" were options, but to plot with the other Yes/No questions, it look slike "Other" and/or "Maybe" were options when they were not.)

7.1 A: 5-level Agree-to-Disagree Scale

Reformat data for these plots. Only Ordinal variables with the agree - disagree scale.

Note to make this all work in one figure I had to make all the levels of the variables consistent

- In Q4, Q5, Q8, there are 5 levels of response ("Strongly disagree","Somewhat disagree","Neither agree nor disagree","Somewhat agree","Strongly agree"), but in Q12 there is a level "Disagree" instead of "Somewhat disagree". To make everything consistent, I changed the second level to "Disagree / Somewhat disagree" for all of these questions.
- Q9 only had 3 levels, but I altered it to have the same 5 levels as the others (but no one can respond to the "Strongly" agree/disagree options as these did not exist)

```
>   OrderA <- c("Strongly disagree","Disagree / Somewhat disagree","Neither agree nor disagree","Somewhat agree","Strongly agree")
>
>   dat.figA <- dat %>%
+     select(Legal, Q4, Q5, Q8, Q9, Q12) %>%

+     ### Need to make the levels of the responses consistent
+     mutate(
+       Q4 = factor(Q4, levels=Order1, labels=OrderA, ordered=TRUE),
+       Q5 = factor(Q5, levels=Order1, labels=OrderA, ordered=TRUE),
+       Q8 = factor(Q8, levels=Order1, labels=OrderA, ordered=TRUE),
+       Q9 = factor(Q9, levels=Order1, labels=OrderA, ordered=TRUE),
+       Q12 = factor(Q12, levels=Order2, labels=OrderA, ordered=TRUE)
```

```
+     ) %>%

+     pivot_longer(cols=c("Q4", "Q5", "Q8","Q9","Q12"),
+                  names_to="Variable",
+                  values_to="Response",
+                  values_drop_na=TRUE) %>%

+     mutate(Label = factor(Variable, levels=c("Q4","Q5","Q8","Q9","Q12"),
+                                   labels=c("A1","A2","A3","A4", "A5")))
```

```
>   ggA <-
+     dat.figA %>%
+       ggplot(aes(Legal)) +
+       geom_bar(aes(fill=Response),position = position_fill(reverse = TRUE))+

+       facet_wrap(~Label, nrow=1)+


+       scale_fill_manual(values=brewer.pal(n=5, name="RdBu"), drop=FALSE, guide = guide_legend(reverse=TRUE))+
+       theme(panel.background=element_blank(),
+             panel.border = element_rect(fill=NA,color="black", linewidth=0.5, linetype="solid")) +
+       theme(strip.background = element_blank())+
+       theme(strip.text.y.right = element_text(angle = 0, hjust=0))+
+       labs(x="",fill="",y="Proportion")+
+       ggtitle("A")
```

```
>   ggA
```

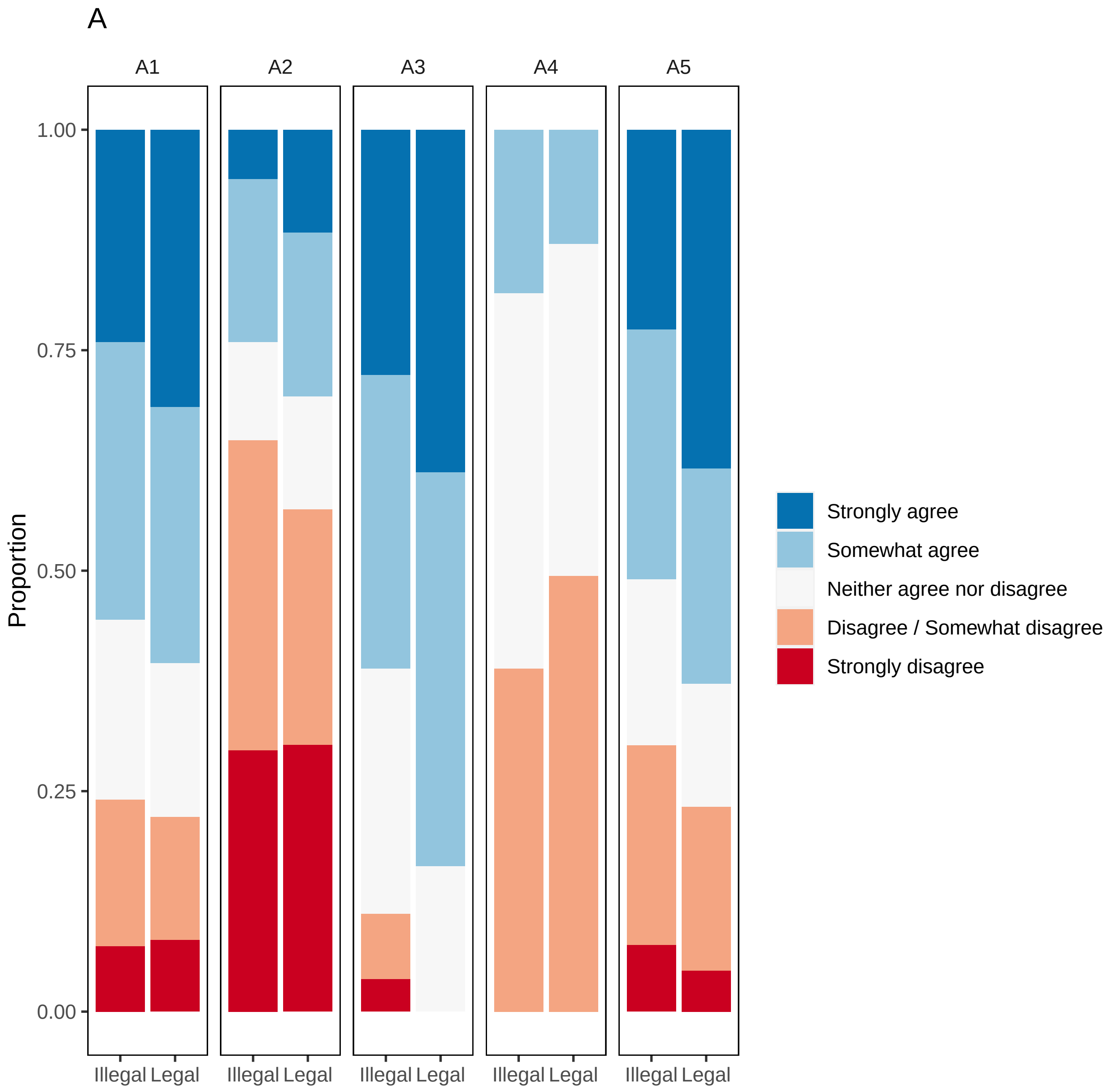
A
A1
A2
A3
A4
A5
Proportion
1.00
0.75
0.50
0.25
0.00
Illegal
Legal
Strongly agree
Somewhat agree
Neither agree nor disagree
Disagree / Somewhat disagree
Strongly disagree

7.2 B: Yes/No (and Maybe/Other)

Note: to put all of these on the same plot, I needed to force consistent scales (that is, make all the questions have the same set of levels). Some had only Yes/No for options, some had Yes/No/Maybe, some had Yes/No/Other. So I forced all 3 to *artificially* have 3 levels: "Yes", "No", and "Maybe/Other".

```
>   OrderB <- c("No","Maybe/Other","Yes")
>
>   dat.figB <- dat %>%
+     select(Legal, Q7, Q11, Q8, Q13, Q14) %>%

+     ### Need to make the levels of the responses consistent
+     mutate(
+       Q7 = factor(Q7, levels=OrderB, labels=OrderB, ordered=TRUE),
+       Q11 = factor(Q11, levels=c("No","Maybe","Yes"), labels=OrderB, ordered=TRUE),
+       Q13 = factor(Q13, levels=c("No","Other","Yes"), labels=OrderB, ordered=TRUE),
+       Q14 = factor(Q14, levels=c("No","Other","Yes"), labels=OrderB, ordered=TRUE),
+     ) %>%

+     pivot_longer(cols=c("Q7", "Q11", "Q13","Q14"),
+                  names_to="Variable",
+                  values_to="Response",
+                  values_drop_na=TRUE) %>%

+     mutate(Label = factor(Variable, levels=c("Q7", "Q11", "Q13","Q14"),
+                                   labels=c("B1","B2","B3","B4")))
```

Extracting color scale

```
>   #display.brewer.pal(5,"RdBu")
>   #Colors.FigB <- brewer.pal(5,"RdBu")[c(1,3,5)]
>
>   #display.brewer.pal(11,"RdBu")
>   #Colors.FigB <- brewer.pal(11,"RdBu")[c(3,6,9)]
>   Colors.FigB <- brewer.pal(11,"RdYlGn")[c(2,6,10)]
```

```
>   ggB <-
+     dat.figB %>%
+       ggplot(aes(Legal)) +
+       geom_bar(aes(fill=Response),position = position_fill(reverse = TRUE))+
+       facet_wrap(~Label, nrow=1)+
+       scale_fill_manual(values=Colors.FigB, drop=FALSE, guide = guide_legend(reverse=TRUE))+
+       theme(panel.background=element_blank(),
+             panel.border = element_rect(fill=NA,color="black", linewidth=0.5, linetype="solid")) +
+       theme(strip.background = element_blank())+
+       theme(strip.text.y.right = element_text(angle = 0, hjust=0))+
+       labs(x="",fill="",y="Proportion")+
+       ggtitle("B")
```

```
>   ggB
```

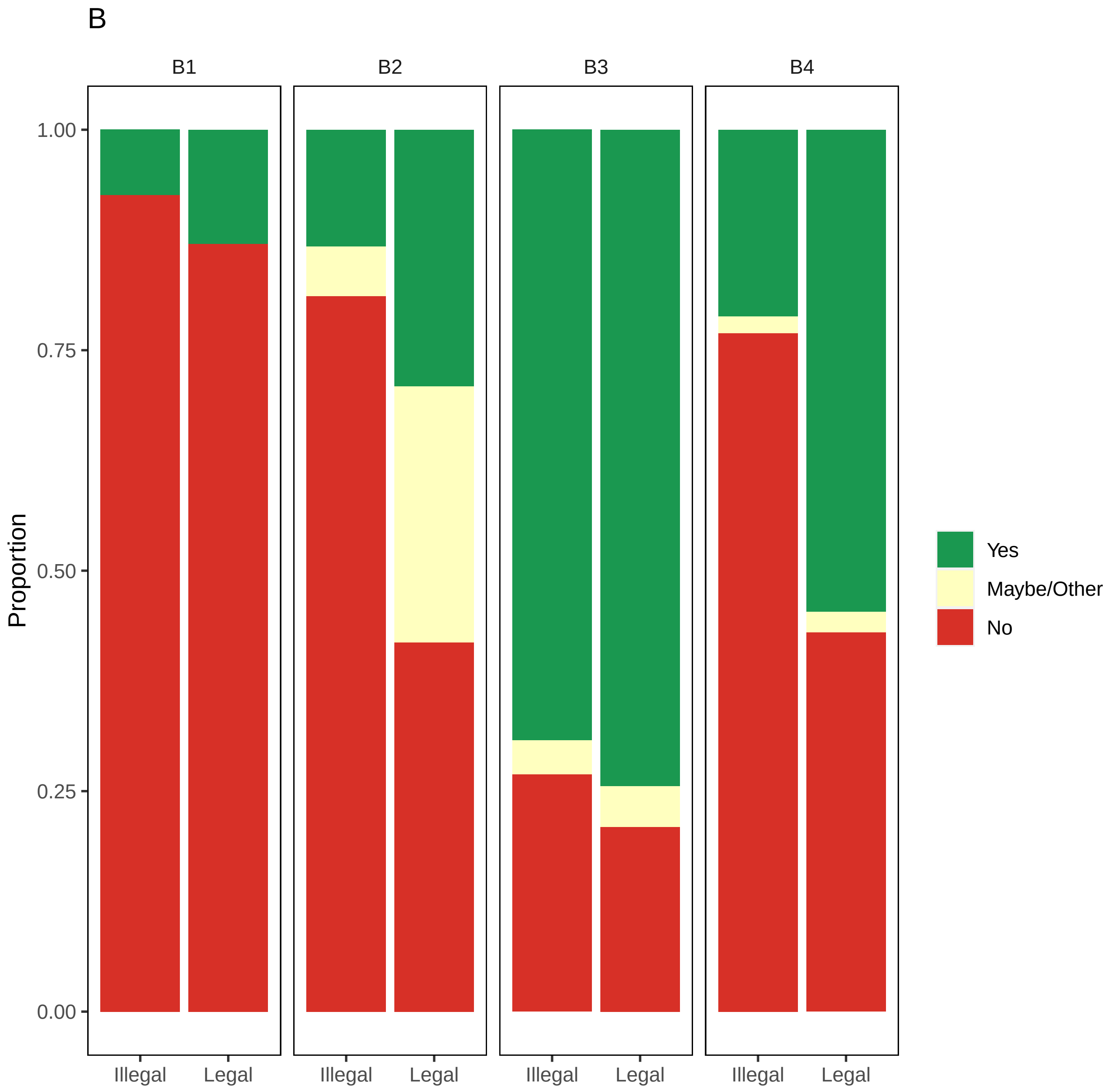
B
B1
B2
B3
B4
Proportion
1.00
0.75
0.50
0.25
0.00
Illegal
Legal
Yes
Maybe/Other
No

7.3 E (Q3-only)

```
> ggE <-
+   dat %>%
+     ggplot(aes(Legal))+
+       geom_bar(aes(fill=Q3),position = position_fill(reverse = TRUE))+
+       scale_fill_discrete(drop=FALSE, guide = guide_legend(reverse=TRUE))+
+       theme(panel.background=element_blank(),
+           panel.border = element_rect(fill=NA,color="black", linewidth=0.5, linetype="solid")) +
+       theme(strip.background = element_blank())+
+       theme(strip.text.y.right = element_text(angle = 0, hjust=0))+
+       labs(x="",fill="",y="Proportion")+
+       ggtitle("E")
```

```
> ggE
```

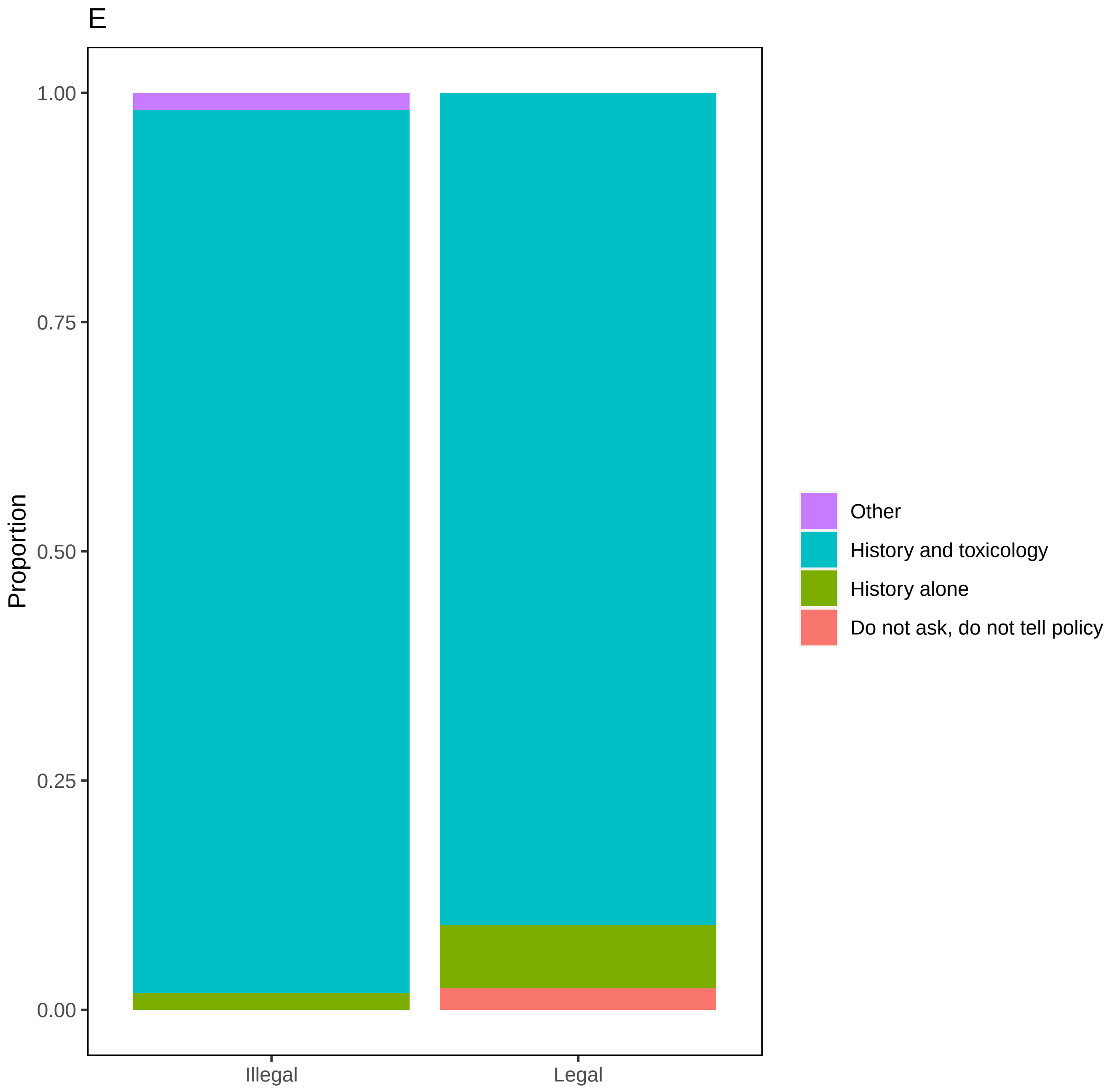
E
Proportion
1.00
0.75
0.50
0.25
0.00
Illegal
Legal
Other
History and toxicology
History alone
Do not ask, do not tell policy

7.4 C (Q6-only)

```
>   ggC <-
+     dat %>% filter(!is.na(Q6)) %>%
+       ggplot(aes(Legal))+
+         geom_bar(aes(fill=Q6),position = position_fill(reverse = TRUE))+
+         scale_fill_brewer(drop=FALSE, guide = guide_legend(reverse=TRUE))+
+         theme(panel.background=element_blank(),
+             panel.border = element_rect(fill=NA,color="black", linewidth=0.5, linetype="solid")) +
+         theme(strip.background = element_blank())+
+         theme(strip.text.y.right = element_text(angle = 0, hjust=0))+
+         labs(x="",fill="",y="Proportion")+
+         ggtitle("C")
```

```
>   ggC
```

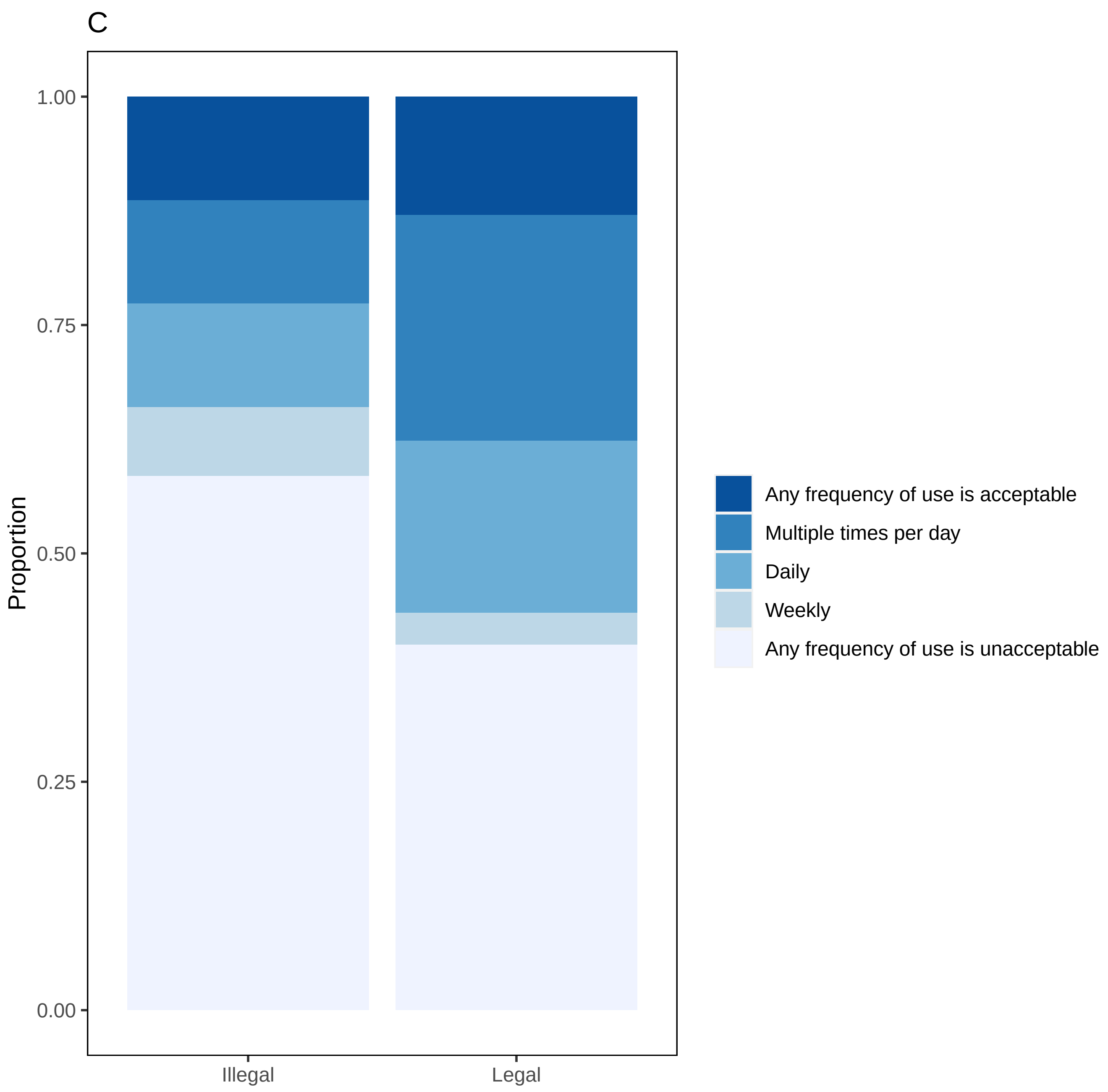
C
Proportion
1.00
0.75
0.50
0.25
0.00
Illegal
Legal
Any frequency of use is acceptable
Multiple times per day
Daily
Weekly
Any frequency of use is unacceptable

7.5 D (Q15-only)

```
>   ggD <-
+     dat %>% filter(!is.na(Q15)) %>%
+       ggplot(aes(Legal))+
+         geom_bar(aes(fill=Q15),position = position_fill(reverse = TRUE))+
+         scale_fill_brewer(palette=5,drop=FALSE, guide = guide_legend(reverse=TRUE))+
+         #scale_fill_grey(,drop=FALSE, guide = guide_legend(reverse=TRUE))+
+         theme(panel.background=element_blank(),
+             panel.border = element_rect(fill=NA,color="black", linewidth=0.5, linetype="solid")) +
+         theme(strip.background = element_blank())+
+         theme(strip.text.y.right = element_text(angle = 0, hjust=0))+
+         labs(x="",fill="",y="Proportion")+
+         ggtitle("D")
```

```
>   ggD
```

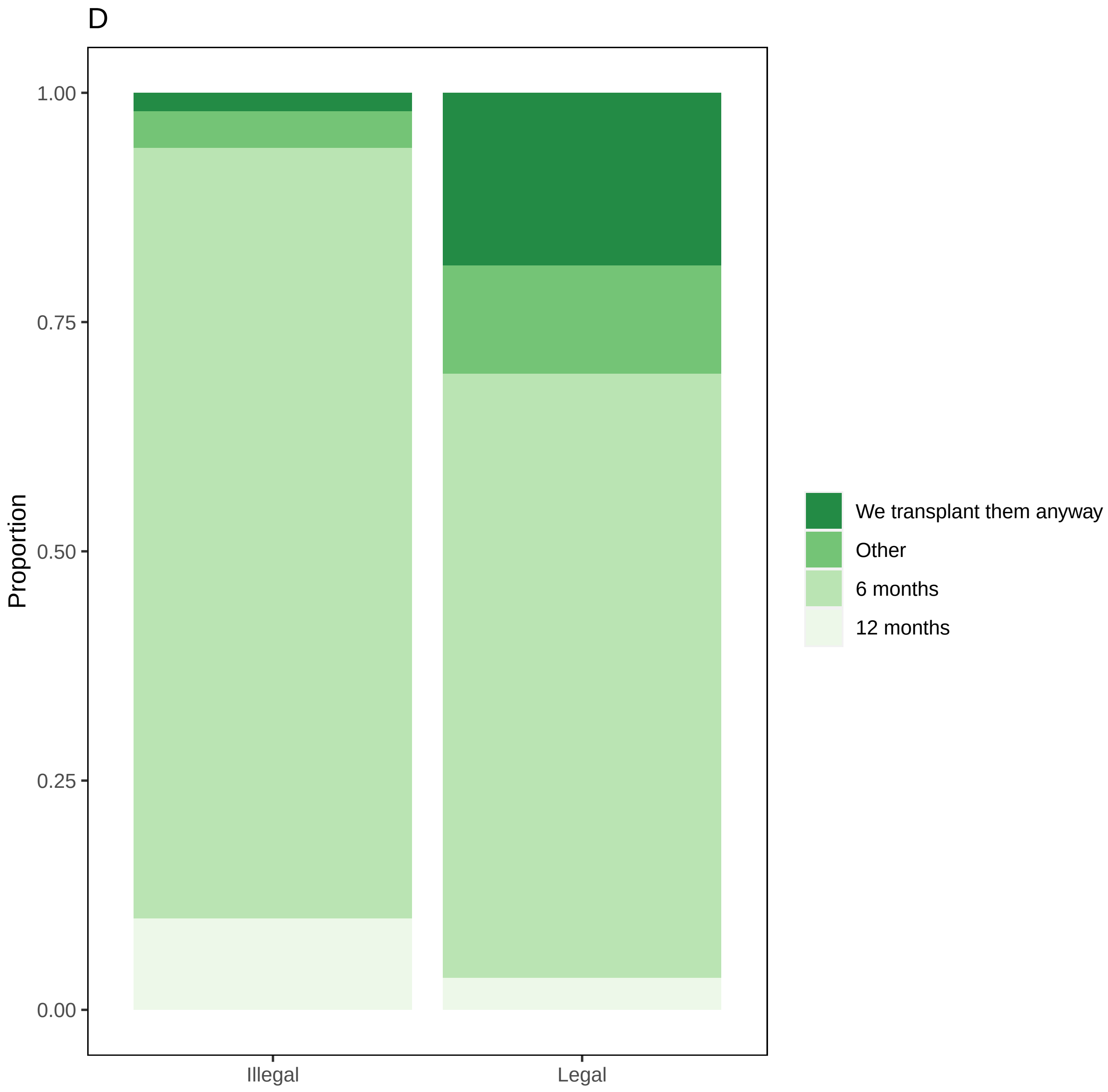
D
1.00
0.75
0.50
0.25
0.00
Proportion
Illegal
Legal
We transplant them anyway
Other
6 months
12 months

7.6 Combine Plots

Output BITMAP file to C:/Users/shaknapp/Indiana University/O365-BreathettStats - IlonzeOnyedika - IlonzeOnyedika/ (Teams directory)

Version 1

```
>   library(gridExtra)



Attaching package:  'gridExtra'


The following object is masked from 'package:dplyr':

    combine


>   bmp(filename = paste0(OUTDIR,RPTNAME,"_ggv1.bmp"),width=18, height=12,units="in", res=300)
>
>         grid.arrange(arrangeGrob(ggA,ggB, ncol=2),
+                 arrangeGrob(ggC, ggD, ggE, ncol=3),
+                 nrow=2)
>
>   dev.off()


pdf
  2
```

Version 2

```
>   library(cowplot)
>   bmp(filename = paste0(OUTDIR,RPTNAME,"_ggv2.bmp"),width=18, height=12,units="in", res=300)
>
>         plot_grid(ggA,ggC,ggB, ggD, ncol=2, nrow=2, rel_widths = c(2/3,1/3))
>
>   dev.off()


pdf
  2
```

Chapter 8

Appendix

8.1 R Session Information

Current Report Generated:

```
>   Sys.time()


[1] "2023-06-20 07:51:11 PDT"
```

The following gives the Session Information for this analysis. This includes the version of R used and the versions of all packages used.

```
>   sessionInfo()


R version 4.2.3 (2023-03-15 ucrt)
Platform: x86_64-w64-mingw32/x64 (64-bit)
Running under: Windows 10 x64 (build 19045)

Matrix products: default

locale:
[1] LC_COLLATE=English_United States.utf8  LC_CTYPE=English_United States.utf8    LC_MONETARY=English_United States.utf8
[4] LC_NUMERIC=C                           LC_TIME=English_United States.utf8

attached base packages:
[1] stats     graphics  grDevices utils     datasets  methods   base

other attached packages:
 [1] cowplot_1.1.1      gridExtra_2.3      RColorBrewer_1.1-3 tidyr_1.3.0        janitor_2.1.0      stringr_1.5.0
 [7] dplyr_1.0.10       lawstat_3.6        ggplot2_3.4.2      xlsx_0.6.5         knitr_1.40

loaded via a namespace (and not attached):
 [1] highr_0.9        pillar_1.9.0     compiler_4.2.3   tools_4.2.3      boot_1.3-28.1    timechange_0.1.1
 [7] lubridate_1.9.0  evaluate_0.18    lifecycle_1.0.3  tibble_3.1.8     gtable_0.3.1     pkgconfig_2.0.3
[13] rlang_1.1.1      cli_3.4.1        DBI_1.1.3        rstudioapi_0.14  Kendall_2.2.1    mvtnorm_1.1-3
[19] xfun_0.39        rJava_1.0-6      withr_2.5.0      generics_0.1.3   xlsxjars_0.6.1   vctrs_0.6.2
[25] grid_4.2.3       tidyselect_1.2.0 snakecase_0.11.0 glue_1.6.2       R6_2.5.1         fansi_1.0.3
[31] Rdpack_2.4       farver_2.1.1     purrr_1.0.1      magrittr_2.0.3   rbibutils_2.2.13 scales_1.2.1
[37] colorspace_2.0-3 labeling_0.4.2   utf8_1.2.2       stringi_1.7.8    munsell_0.5.0
```

8.2 Citations

8.2.1 Citations (etc.) for Brunner-Munzel Test

Brunner, E. and U. Munzel (2000). "The nonparametric Behrens-Fisher Problem: Asymptotic Theory and a small sample approximation." Biometrical Journal 42(1): 17-25.

Neuber, Karin and Edgar Brunner. 2007. A studentized permutation test for the non-parametric BehrensFisher problem. Computational Statistics Data Analysis. Volume 51, Issue 10, 15 June 2007, Pages 5192-5204. `https://doi.org/10.1016/j.csda.2006.05.024`

The function used to do the Brunner-Munzel test is in the R package 'lawstat' (citation below)

```
To cite package 'lawstat' in publications use:

  Gastwirth J, Gel Y, Hui W, Lyubchich V, Miao W, Noguchi K (2023). _lawstat: Tools for Biostatistics,
  Public Policy, and Law_. R package version 3.6, <https://CRAN.R-project.org/package=lawstat>.

A BibTeX entry for LaTeX users is

  @Manual{,
    title = {lawstat: Tools for Biostatistics, Public Policy, and Law},
    author = {Joseph L. Gastwirth and Yulia R. Gel and W. L. Wallace Hui and Vyacheslav Lyubchich and Weiwen Miao and Kimihiro Noguchi},
    year = {2023},
    note = {R package version 3.6},
    url = {https://CRAN.R-project.org/package=lawstat},
  }
```

Function documentation: `https://www.rdocumentation.org/packages/lawstat/versions/3.4/topics/brunner.munzel.test`

8.2.2 Citations for R and R-packages

Citation for R

```
To cite R in publications use:

  R Core Team (2023). R: A language and environment for statistical computing. R Foundation for Statistical
  Computing, Vienna, Austria. URL https://www.R-project.org/.

A BibTeX entry for LaTeX users is

  @Manual{,
    title = {R: A Language and Environment for Statistical Computing},
    author = {{R Core Team}},
    organization = {R Foundation for Statistical Computing},
    address = {Vienna, Austria},
    year = {2023},
    url = {https://www.R-project.org/},
  }

We have invested a lot of time and effort in creating R, please cite it when using it for data analysis.
See also 'citation("pkgname")' for citing R packages.
```

Citation for R packages (libraries) used

```
======================================================================
package: cowplot
----------------------------------------------------------------------

To cite package ’cowplot’ in publications use:

  Wilke C (2020). _cowplot: Streamlined Plot Theme and Plot Annotations for ’ggplot2’_. R package version
  1.1.1, <https://CRAN.R-project.org/package=cowplot>.

A BibTeX entry for LaTeX users is

  @Manual{,
    title = {cowplot: Streamlined Plot Theme and Plot Annotations for ’ggplot2’},
    author = {Claus O. Wilke},
    year = {2020},
    note = {R package version 1.1.1},
    url = {https://CRAN.R-project.org/package=cowplot},
  }




======================================================================
package: gridExtra
----------------------------------------------------------------------

To cite package ’gridExtra’ in publications use:

  Auguie B (2017). _gridExtra: Miscellaneous Functions for "Grid" Graphics_. R package version 2.3,
  <https://CRAN.R-project.org/package=gridExtra>.

A BibTeX entry for LaTeX users is

  @Manual{,
    title = {gridExtra: Miscellaneous Functions for "Grid" Graphics},
    author = {Baptiste Auguie},
    year = {2017},
    note = {R package version 2.3},
    url = {https://CRAN.R-project.org/package=gridExtra},
  }




======================================================================
package: RColorBrewer
----------------------------------------------------------------------

To cite package ’RColorBrewer’ in publications use:

  Neuwirth E (2022). _RColorBrewer: ColorBrewer Palettes_. R package version 1.1-3,
  <https://CRAN.R-project.org/package=RColorBrewer>.

A BibTeX entry for LaTeX users is

  @Manual{,
    title = {RColorBrewer: ColorBrewer Palettes},
    author = {Erich Neuwirth},
    year = {2022},
    note = {R package version 1.1-3},
    url = {https://CRAN.R-project.org/package=RColorBrewer},
  }




======================================================================
package: tidyr
```

```
----------------------------------------------------------------------

To cite package 'tidyr' in publications use:

  Wickham H, Vaughan D, Girlich M (2023). _tidyr: Tidy Messy Data_. R package version 1.3.0,
  <https://CRAN.R-project.org/package=tidyr>.

A BibTeX entry for LaTeX users is

  @Manual{,
    title = {tidyr: Tidy Messy Data},
    author = {Hadley Wickham and Davis Vaughan and Maximilian Girlich},
    year = {2023},
    note = {R package version 1.3.0},
    url = {https://CRAN.R-project.org/package=tidyr},
  }



======================================================================
package: janitor
----------------------------------------------------------------------

To cite package 'janitor' in publications use:

  Firke S (2021). _janitor: Simple Tools for Examining and Cleaning Dirty Data_. R package version 2.1.0,
  <https://CRAN.R-project.org/package=janitor>.

A BibTeX entry for LaTeX users is

  @Manual{,
    title = {janitor: Simple Tools for Examining and Cleaning Dirty Data},
    author = {Sam Firke},
    year = {2021},
    note = {R package version 2.1.0},
    url = {https://CRAN.R-project.org/package=janitor},
  }



======================================================================
package: stringr
----------------------------------------------------------------------

To cite package 'stringr' in publications use:

  Wickham H (2022). _stringr: Simple, Consistent Wrappers for Common String Operations_. R package version
  1.5.0, <https://CRAN.R-project.org/package=stringr>.

A BibTeX entry for LaTeX users is

  @Manual{,
    title = {stringr: Simple, Consistent Wrappers for Common String Operations},
    author = {Hadley Wickham},
    year = {2022},
    note = {R package version 1.5.0},
    url = {https://CRAN.R-project.org/package=stringr},
  }



======================================================================
package: dplyr
----------------------------------------------------------------------
```

```
To cite package 'dplyr' in publications use:

  Wickham H, Franois R, Henry L, Mller K (2022). _dplyr: A Grammar of Data Manipulation_. R package
  version 1.0.10, <https://CRAN.R-project.org/package=dplyr>.

A BibTeX entry for LaTeX users is

  @Manual{,
    title = {dplyr: A Grammar of Data Manipulation},
    author = {Hadley Wickham and Romain Franois and Lionel Henry and Kirill Mller},
    year = {2022},
    note = {R package version 1.0.10},
    url = {https://CRAN.R-project.org/package=dplyr},
  }




======================================================================
package: lawstat
----------------------------------------------------------------------

To cite package 'lawstat' in publications use:

  Gastwirth J, Gel Y, Hui W, Lyubchich V, Miao W, Noguchi K (2023). _lawstat: Tools for Biostatistics,
  Public Policy, and Law_. R package version 3.6, <https://CRAN.R-project.org/package=lawstat>.

A BibTeX entry for LaTeX users is

  @Manual{,
    title = {lawstat: Tools for Biostatistics, Public Policy, and Law},
    author = {Joseph L. Gastwirth and Yulia R. Gel and W. L. Wallace Hui and Vyacheslav Lyubchich and Weiwen Miao and Kimihiro Noguchi},
    year = {2023},
    note = {R package version 3.6},
    url = {https://CRAN.R-project.org/package=lawstat},
  }




======================================================================
package: ggplot2
----------------------------------------------------------------------

To cite ggplot2 in publications, please use

  H. Wickham. ggplot2: Elegant Graphics for Data Analysis. Springer-Verlag New York, 2016.

A BibTeX entry for LaTeX users is

  @Book{,
    author = {Hadley Wickham},
    title = {ggplot2: Elegant Graphics for Data Analysis},
    publisher = {Springer-Verlag New York},
    year = {2016},
    isbn = {978-3-319-24277-4},
    url = {https://ggplot2.tidyverse.org},
  }




======================================================================
package: xlsx
----------------------------------------------------------------------

To cite package 'xlsx' in publications use:
```

```
  Dragulescu A, Arendt C (2020). _xlsx: Read, Write, Format Excel 2007 and Excel 97/2000/XP/2003 Files_. R
  package version 0.6.5, <https://CRAN.R-project.org/package=xlsx>.

A BibTeX entry for LaTeX users is

  @Manual{,
    title = {xlsx: Read, Write, Format Excel 2007 and Excel 97/2000/XP/2003 Files},
    author = {Adrian Dragulescu and Cole Arendt},
    year = {2020},
    note = {R package version 0.6.5},
    url = {https://CRAN.R-project.org/package=xlsx},
  }




======================================================================
package: knitr
----------------------------------------------------------------------

To cite the 'knitr' package in publications use:

  Yihui Xie (2022). knitr: A General-Purpose Package for Dynamic Report Generation in R. R package version
  1.40.

  Yihui Xie (2015) Dynamic Documents with R and knitr. 2nd edition. Chapman and Hall/CRC. ISBN
  978-1498716963

  Yihui Xie (2014) knitr: A Comprehensive Tool for Reproducible Research in R. In Victoria Stodden,
  Friedrich Leisch and Roger D. Peng, editors, Implementing Reproducible Computational Research. Chapman
  and Hall/CRC. ISBN 978-1466561595

To see these entries in BibTeX format, use 'print(<citation>, bibtex=TRUE)', 'toBibtex(.)', or set
'options(citation.bibtex.max=999)'.


======================================================================
package: stats
----------------------------------------------------------------------

The 'stats' package is part of R.  To cite R in publications use:

  R Core Team (2023). R: A language and environment for statistical computing. R Foundation for Statistical
  Computing, Vienna, Austria. URL https://www.R-project.org/.

A BibTeX entry for LaTeX users is

  @Manual{,
    title = {R: A Language and Environment for Statistical Computing},
    author = {{R Core Team}},
    organization = {R Foundation for Statistical Computing},
    address = {Vienna, Austria},
    year = {2023},
    url = {https://www.R-project.org/},
  }

We have invested a lot of time and effort in creating R, please cite it when using it for data analysis.
See also 'citation("pkgname")' for citing R packages.


======================================================================
package: graphics
----------------------------------------------------------------------

The 'graphics' package is part of R.  To cite R in publications use:

  R Core Team (2023). R: A language and environment for statistical computing. R Foundation for Statistical
```

```
  Computing, Vienna, Austria. URL https://www.R-project.org/.

A BibTeX entry for LaTeX users is

  @Manual{,
    title = {R: A Language and Environment for Statistical Computing},
    author = {{R Core Team}},
    organization = {R Foundation for Statistical Computing},
    address = {Vienna, Austria},
    year = {2023},
    url = {https://www.R-project.org/},
  }

We have invested a lot of time and effort in creating R, please cite it when using it for data analysis.
See also 'citation("pkgname")' for citing R packages.


======================================================================
package: grDevices
----------------------------------------------------------------------

The 'grDevices' package is part of R.  To cite R in publications use:

  R Core Team (2023). R: A language and environment for statistical computing. R Foundation for Statistical
  Computing, Vienna, Austria. URL https://www.R-project.org/.

A BibTeX entry for LaTeX users is

  @Manual{,
    title = {R: A Language and Environment for Statistical Computing},
    author = {{R Core Team}},
    organization = {R Foundation for Statistical Computing},
    address = {Vienna, Austria},
    year = {2023},
    url = {https://www.R-project.org/},
  }

We have invested a lot of time and effort in creating R, please cite it when using it for data analysis.
See also 'citation("pkgname")' for citing R packages.


======================================================================
package: utils
----------------------------------------------------------------------

The 'utils' package is part of R.  To cite R in publications use:

  R Core Team (2023). R: A language and environment for statistical computing. R Foundation for Statistical
  Computing, Vienna, Austria. URL https://www.R-project.org/.

A BibTeX entry for LaTeX users is

  @Manual{,
    title = {R: A Language and Environment for Statistical Computing},
    author = {{R Core Team}},
    organization = {R Foundation for Statistical Computing},
    address = {Vienna, Austria},
    year = {2023},
    url = {https://www.R-project.org/},
  }

We have invested a lot of time and effort in creating R, please cite it when using it for data analysis.
See also 'citation("pkgname")' for citing R packages.


======================================================================
package: datasets
```

```
----------------------------------------------------------------------

The 'datasets' package is part of R.  To cite R in publications use:

  R Core Team (2023). R: A language and environment for statistical computing. R Foundation for Statistical
  Computing, Vienna, Austria. URL https://www.R-project.org/.

A BibTeX entry for LaTeX users is

  @Manual{,
    title = {R: A Language and Environment for Statistical Computing},
    author = {{R Core Team}},
    organization = {R Foundation for Statistical Computing},
    address = {Vienna, Austria},
    year = {2023},
    url = {https://www.R-project.org/},
  }

We have invested a lot of time and effort in creating R, please cite it when using it for data analysis.
See also 'citation("pkgname")' for citing R packages.


======================================================================
package: methods
----------------------------------------------------------------------

The 'methods' package is part of R.  To cite R in publications use:

  R Core Team (2023). R: A language and environment for statistical computing. R Foundation for Statistical
  Computing, Vienna, Austria. URL https://www.R-project.org/.

A BibTeX entry for LaTeX users is

  @Manual{,
    title = {R: A Language and Environment for Statistical Computing},
    author = {{R Core Team}},
    organization = {R Foundation for Statistical Computing},
    address = {Vienna, Austria},
    year = {2023},
    url = {https://www.R-project.org/},
  }

We have invested a lot of time and effort in creating R, please cite it when using it for data analysis.
See also 'citation("pkgname")' for citing R packages.


======================================================================
package: base
----------------------------------------------------------------------

To cite R in publications use:

  R Core Team (2023). R: A language and environment for statistical computing. R Foundation for Statistical
  Computing, Vienna, Austria. URL https://www.R-project.org/.

A BibTeX entry for LaTeX users is

  @Manual{,
    title = {R: A Language and Environment for Statistical Computing},
    author = {{R Core Team}},
    organization = {R Foundation for Statistical Computing},
    address = {Vienna, Austria},
    year = {2023},
    url = {https://www.R-project.org/},
  }

We have invested a lot of time and effort in creating R, please cite it when using it for data analysis.
```

See also 'citation("pkgname")' for citing R packages.
